# ﻿Phylogeny and classification of the Australasian and Indomalayan mimosoid legumes *Archidendron* and *Archidendropsis* (Leguminosae, subfamily Caesalpinioideae, mimosoid clade)

**DOI:** 10.3897/phytokeys.205.79381

**Published:** 2022-08-22

**Authors:** Gillian K. Brown, Javier Aju, Michael J. Bayly, Daniel J. Murphy, Todd G. B. McLay

**Affiliations:** 1 School of Biosciences, University of Melbourne, Parkville, Victoria, 3010, Australia University of Melbourne Parkville Australia; 2 Queensland Herbarium, Department of Environment and Science, Toowong, Queensland, 4066, Australia Queensland Herbarium, Department of Environment and Science Toowong Australia; 3 Departmento de Biología, Universidad del Valle de Guatemala, Guatemala, Guatemala Universidad del Valle de Guatemala Guatemala Guatemala; 4 National Herbarium of Victoria, Royal Botanic Gardens Victoria, South Yarra, Victoria, 3141, Australia National Herbarium of Victoria, Royal Botanic Gardens Victoria South Yarra Australia

**Keywords:** Fabaceae, ingoid clade, legumes, low copy nuclear gene, Malesia, phylogeny, targeted amplicon sequencing

## Abstract

The morphologically variable genus *Archidendron* is the second largest mimosoid legume genus from the Indomalayan-Australasian region, yet it has not been well represented in phylogenetic studies. Phylogenies that have included multiple representatives of *Archidendron* suggest it may not be monophyletic, and the same applies to *Archidendropsis*, another understudied genus of the Archidendron clade. The most comprehensive phylogeny of *Archidendron* and *Archidendropsis* to date is presented, based on four nuclear markers (ITS, ETS, SHMT and RBPCO). Exemplars from all genera of the wider Archidendron clade are sampled, including representatives of all series within *Archidendron* and the two subgenera of *Archidendropsis*. Our results confirm that *Archidendron* and *Archidendropsis* are not monophyletic. Within *Archidendron*, only one series (ser. Ptenopae) is resolved as monophyletic and species of *Archidendron* are divided into two primarily geographic lineages. One clade is distributed in western Malesia and mainland Asia and includes most representatives of series *Clypeariae*, while the other is mostly restricted to eastern Malesia and Australia and includes representatives of the seven other series plus two samples of series *Clypeariae*. No taxonomic changes are made for *Archidendron* due to the high level of topological uncertainty and the lack of discrete macromorphological characters separating these two lineages. Each of the two subgenera of *Archidendropsis* is monophyletic but they are not closely related. A new genus endemic to Queensland (Australia), *Heliodendron* Gill.K. Br. & Bayly, **gen. nov.**, is described for the former Archidendropsissubg.Basaltica, and combinations for its three species are proposed: *Heliodendronbasalticum* (F. Muell.) Gill.K. Br. & Bayly, **comb. nov.**, *Heliodendronthozetianum* (F. Muell.) Gill.K. Br. & Bayly, **comb. nov.**, and *Heliodendronxanthoxylon* (C.T. White & W.D. Francis) Gill.K. Br. & Bayly, **comb. nov.**

## ﻿Introduction

The classification of mimosoid legumes has been significantly transformed in the past 20 years since the first comprehensive molecular phylogeny of the then subfamily Mimosoideae (Luckow et al. 2003). Understanding of relationships within the mimosoid legumes has improved through studies at generic, regional, alliance, subfamilial and familial levels (see references in Legume Phylogeny Working Group 2017; Koenen et al. 2020; Ringelberg et al. 2022). In the comprehensive phylogeny and revision of the legume family (Leguminosae or Fabaceae), the mimosoid legumes formed a clade nested within the re-circumscribed subfamily Caesalpinioideae (Legume Phylogeny Working Group 2017). Recent phylogenomic data have sufficiently enhanced resolution to enable recognition of several clades within subfamily Caesalpinioideae, including the mimosoid, core mimosoid and ingoid clades (Koenen et al. 2020; Ringelberg et al. 2022). However, within these clades some large genera, such as *Archidendron* F. Muell. and allies have remained under-studied relative to *Acacia* Mill. s.l. and many Neotropical ingoid genera and groups (e.g. Murphy et al. 2010; de Souza et al. 2013; Iganci et al. 2016; Miller et al. 2017; Ferm et al. 2019; Comben et al. 2020).

The two largest mimosoid genera from the Indomalayan-Australasian region are *Acacia* and *Archidendron*. These are placed in the Archidendron clade (sensu Koenen et al. 2020), along with *Archidendropsis* I.C. Nielsen, *Falcataria* (I.C. Nielsen) Barneby & J.W. Grimes, *Pararchidendron* I.C. Nielsen, *Paraserianthes* I.C. Nielsen, *Serianthes* Benth. and *Wallaceodendron* Koord. The Archidendron clade is biogeographically distinct within the mimosoid legumes, being primarily restricted to the Indomalayan and Australasian regions, and has been given several names over the years to reflect this: the Australian & SE Asian Ingeae clade (Brown et al. 2008) and the Australo-Malesian mimosoids (Brown et al. 2011). Within the Archidendron clade, *Pararchidendron*, *Paraserianthes* and *Wallaceodendron* are monotypic, and three of the other five genera (*Acacia* s.s., *Falcataria*, and *Serianthes*) are well documented as monophyletic based on morphological and genetic data (Chappill and Maslin 1995; Miller and Bayer 2001; Luckow et al. 2003; Brown et al. 2008, 2011; Murphy et al. 2010; Demeulenaere et al. 2022; Ringelberg et al. 2022). However, *Archidendron* has been suggested to be paraphyletic (Brown et al. 2008, 2011; Iganci et al. 2016; Demeulenaere et al. 2022; Ringelberg et al. 2022), as has *Archidendropsis* (Demeulenaere et al. 2022; Ringelberg et al. 2022).

**Figure 1.**
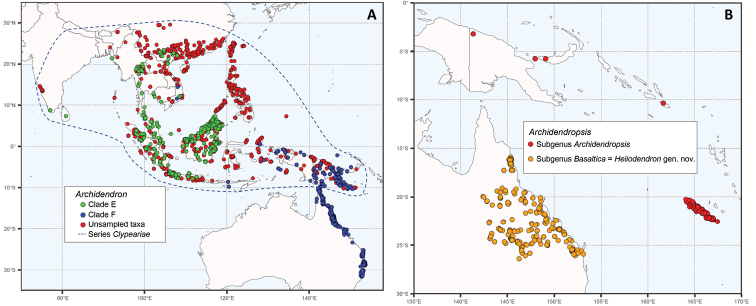
Distribution maps of the genera *Archidendron* and *Archidendropsis*. The maps are based on quality-controlled species-level digitised herbarium specimens from GBIF (www.gbif.org) (Ringelberg et al. 2022). Maps were created using R packages ggplot2 (Wickham 2016), sf (Pebesma 2018), and rnaturalearth (South 2017) **A***Archidendron*. Species distributions are coloured according to the ncDNA phylogeny clades (Fig. 2) except for *A.clypearia*: Clade E (Clypeariae clade) = green dots; clade F (Archidendron s.s. clade) = blue dots; species not sampled for the phylogeny = red dots. *Archidendronclypearia* is widespread and falls in both clades E and F, so for this species locations of samples in the ncDNA phylogeny are coloured according to their clade and all other records of this species are coloured red. The overall distribution of series *Clypeariae* is shown by a blue dashed line **B***Archidendropsis*. All species that belong to subg. Archidendropsis are coloured red and those in subg. Basaltica (= *Heliodendron* gen. nov.) are coloured orange.

*Archidendron* is the second largest genus in this clade after *Acacia*, with 99 described species and an additional 20 putative species that are poorly known due to limited collections or destroyed types (Nielsen et al. 1984b; Cowan 1998; Wu and Nielsen 2010; Dash and Sanjappa 2011). They are small to medium-sized trees found in lowland and montane tropical and subtropical rainforests of the Australo-Malesian and Pacific regions, distributed from Kerala (southern India) and Sri Lanka in the west, to the Solomon Islands in the east; and from Taiwan and the Ryukyu Islands in the north, to Australia in the south (Fig. 1; Nielsen et al. 1984b, 1984a). In the 1970s and 1980s, an extensive revision of the Australo-Malesian and Pacific Ingeae was undertaken (Nielsen 1979, 1981, 1982; Nielsen et al. 1983b, 1983a, 1984b) and *Archidendron* was expanded based on evidence from wood, pollen, seed and inflorescence characteristics to include species previously referred by Kostermans (1954) to the genera *Abarema* Pittier, *Cylindrokelupha* Kosterm., *Morolobium* Kosterm., *Paralbizzia* Kosterm., *Zygia* P. Browne, and by Bentham (1875) to Pithecellobiumsect.Clypearia sensu Benth. (Baretta-Kuipers 1981; Nielsen et al. 1984b; Nielsen 1992). *Archidendron* now includes unarmed trees or shrubs with bipinnate leaves, mostly opposite leaflets, extrafloral nectaries, and wood anatomy of strictly uniseriate rays and abundant parenchyma with a banded distribution (Nielsen et al. 1984b).

*Archidendron* is morphologically variable especially in leaf, inflorescence, flower, and pod characteristics, and has been divided into eight series (Nielsen et al. 1984b): *Clypeariae* (Benth.) I.C. Nielsen, *Archidendron*, *Calycinae* I.C. Nielsen, *Bellae* I.C. Nielsen, *Ptenopae* I.C. Nielsen, *Pendulosae* (Mohlenbr.) I.C. Nielsen, *Stipulatae* (Mohlenbr.) I.C. Nielsen and *Morolobiae* (Kosterm.) I.C. Nielsen. The largest series, *Clypeariae* (ca. 51 species) is distributed in mainland southeast Asia, western Malesia, and the Philippines, with only a few species found further east (Fig. 1A). This series is well defined by the absence of stipules and flowers that generally have one carpel per ovary that is often stipitate (Nielsen et al. 1984b). The second largest series, *Archidendron* (ca. 15 species), is found in eastern Malesia and Australia and is defined by the presence of stipules and stipular glands. Four of the series are largely confined to the island of New Guinea (Nielsen et al. 1984b): series *Calycinae* (3 species) with strongly ribbed inflated calyces, cauliflorous racemes and sessile ovaries; series *Bellae* (4 species) with large woody pods without overgrown seeds and cauliflorous paniculate inflorescences; series *Ptenopae* (2 species), which is defined by the presence of two-winged rachises and pinnae; series *Pendulosae* (3 species) have inflorescences with lax racemes (Nielsen et al. 1984a). Series *Stipulatae* (ca. 14 species) are found in New Guinea, the Moluccas, and Queensland (Australia) and have floral bracts with extra floral nectaries, stipular glands and cauliflorous branched racemes (Nielsen et al. 1984b). The three species of series *Morolobiae* have unifoliolate pinnae, and racemose inflorescences with flowers with single, sessile ovaries, and are disjunctly distributed: *A.monopterum* (Kosterm.) I.C. Nielsen in Halmahera (North Maluku Islands, Indonesia), *A.whitei* I.C. Nielsen in northern Queensland (Australia) and *A.muellerianum* (Maiden & R.T. Baker) I.C. Nielsen in northern New South Wales (Australia) (Nielsen et al. 1984b).

Prior to resolution of the Archidendron clade, the genus *Archidendron* was suggested to be related to taxa of the Inga-alliance (Barneby and Grimes 1996; Lewis and Rico Arce 2005) or to other Old World genera, such as *Archidendropsis*, *Falcataria*, *Pararchidendron*, *Paraserianthes* and *Serianthes* (Baretta-Kuipers 1981; Nielsen et al. 1984a; Nielsen 1992). *Archidendron* has not been well represented in molecular phylogenies to date with only ten of the 99 species and four of the eight series (*Archidendron*, *Clypeariae*, *Morolobiae* and *Ptenopae*) included in any one study. In all studies, samples of series *Clypeariae* are placed distantly from the other series (Brown et al. 2008, 2011; Iganci et al. 2016; Koenen et al. 2020; Demeulenaere et al. 2022; Ringelberg et al. 2022).

The genus *Archidendropsis* includes 14 species from New Caledonia, the Solomon Islands, New Britain, Papua New Guinea and Australia (Fig. 1B), with all species endemic to their respective region (Nielsen et al. 1983a). Species of *Archidendropsis* have winged, thin-walled seeds lacking a pleurogram (a mark or depression on both sides of the seed coat; Rodrigues-Junior et al. 2021) and are placed in two subgenera based on pollen and inflorescence characteristics. Species of subgenus Basaltica I.C. Nielsen are restricted to Australia, have smaller polyads (55–60 μm) and globular inflorescences, while species of subgenus Archidendropsis are not found in Australia, have larger polyads (80–120 μm) and flowers arranged in spicate racemes. Like *Archidendron*, *Archidendropsis* has been poorly represented in molecular phylogenies with only one or two of the 14 species included in any one study (Brown et al. 2008, 2011; Ferm et al. 2019; Koenen et al. 2020; Demeulenaere et al. 2022; Ringelberg et al. 2022). Only two studies have included representatives of each of the subgenera and in both, *Archidendropsis* is not resolved as monophyletic (Demeulenaere et al. 2022; Ringelberg et al. 2022).

This study aims to test the monophyly of the genera *Archidendron* and *Archidendropsis* and investigate phylogenetic relationships within the large genus *Archidendron* to test the monophyly of its infrageneric series.

## ﻿Materials and methods

### ﻿Taxon sampling and DNA isolation

A total of 87 accessions were sampled, representing 43 species of *Archidendron* (68 accessions), five species of *Archidendropsis* (six accessions) and nine species (11 accessions) of the other genera in the Archidendron clade; two species of Old World *Albizia* Durazz. were included as outgroups (Table 1). In total 43% of the species of *Archidendron* were sampled including representatives of all eight series. Both subgenera of *Archidendropsis* were sampled covering 36% of species in the genus. Samples were collected in the field and from herbarium specimens sourced from AAU, BISH, BRI, CANB, CNS, KEP, KUN, L, NY, MEL and MELU (herbarium codes as per Thiers, updated continuously).

**Table 1.** Linked data table of specimens sampled for phylogeny. Specimen accession number linking herbarium specimen to sample ID, taxon name with authorities, locality information and geocode (where available) as provided on the specimen/database. GenBank numbers are provided for each marker and where multiple alleles were identified for a specimen, the two GenBank numbers are separated by a semi colon. If the marker was not successfully sequenced for a particular specimen, then the GenBank field is left blank.

Preserved specimen	Associated sequences							Taxon name/MOTU	Sample ID	Location	
Specimen code (InstCode and/or CollCode + Catalogue #)	SHMT	RBPCO	ITS	ETS	trnK	trnV	psbD			Geolocation name / locality	GPS Coordinates
MEL 2294706A			OM286906	OM286992	ON013654			*Acaciabaueri* Benth.	*Z176*	Great Sandy National Park, Fraser Island, Woralie track to Moon Point. Queensland, Australia	153°11'55"E, 25°11'38"S
MELU GB309b	OM984488	OM390190; OM390191	OM286907	OM286993	ON013655	ON101510	OM984574	*Acaciamyrtifolia* (Sm.) Willd.	*JA150*	0.7km north of Playford Highway on Snug Bay Rd, Kangaroo Island, South Australia	136°52'51.8"E, 35°46'30.2"S
CANB 864530.1	OM984489		OM286908	OM286994	ON013656	ON101511	OM984575	*Albizialebbeck* (L.) Benth.	*JA137*	Alva, NE of Ayr, Queensland, Australia	147°28'52"E, 19°27'11"S
MEL 2391890A	OM984490		OM286909	OM286995	ON013657	ON101512	OM984576	*Albiziaretusa* Benth.	*Z106*	Atherton Arboretum. Tag #96. Queensland, Australia	145°29'8.6"E, 17°15'31.4"S
KUN0599506	OM984491		OM286910	OM286996	ON013658	ON101513	OM984577	*Archidendronalternifoliolatum* (T.L.Wu) I.C.Nielsen	*JA25*	China	100.85°E, 24.5667°N
BRI AQ0380081	OM984492; OM984493		OM286911	OM286997	ON013659	ON101514		*Archidendronarborescens* (Kosterm.) I.C.Nielsen	*JA36*	Papua New Guinea, Western Fly; Kwinja Lakes area of the Middle Fly River	141°41'33.987"E, 7°45'24.772S
KUN0599551	OM984494; OM984495		OM286912	OM286998	ON013660	ON101515		*Archidendronbalansae* (Oliv.) I.C.Nielsen	*JA26*	China	
AAU D.McKey92-9	OM984496; OM984497	OM390192	OM286913	OM286999	ON013661	ON101516	OM984578	*Archidendronbigeminum* (L.) I.C.Nielsen	*JA14*	Sinharaja Forest, SW Sri Lanka	80°35'23"E, 6°21'17"N
AAU Balgooy6063	OM984498; OM984499	OM390193	OM286914	OM287000	ON013662	ON101517	OM984579	*Archidendronborneense* (Benth.) I.C.Nielsen	*JA70*	Tanah Merah, Kalimantan Timur	117°‘E, 1°‘S
KEP FRI53789	OM984500; OM984501		OM286915	OM287001	ON013663	ON101518	OM984580	*Archidendronbubalinum* (Jack) I.C.Nielsen	*JA22*	Pahang, Temerloh, Tasik Bera, Kg. Patihir, Malaysia	102.4167°E, 3.8167°N
CANB 730419.1		OM390194; OM390195	OM286916	OM287002	ON013664	ON101519		*Archidendroncalliandrum* de Wit	*JA109*	Ambunti District, Waskut Hills, spur ridge NW of Musapien bivouac. East Sepik, PNG	142°43'55"E, 4°10'36"S
CANB 211609.1	OM984502		OM286917	OM287003	ON013665	ON101520	OM984581	*Archidendroncalycinum* Pulle	*JA129*	Saw Mountains, near junction of Tauri and Kapau Rivers. Gulf Province, PNG	146°8'E, 7°47'S
AAU L.Averyanov4481	OM984503		OM286918	OM287004	ON013666	ON101521	OM984582	*Archidendronchevalieri* (Kosterm.) I.C.Nielsen	*JA71*	Bi Dup ridge, Vietnam	108°39'E, 12°6'N
AAU I.Nielsen26	OM984504		OM286919	OM287005	ON013667	ON101522	OM984583	*Archidendronclypearia* (Jack) I.C.Nielsen	*JA16*	Gunung Mulu National Park, Sarawak	114°55'E, 4°05'N
AAU H.M.Christensen38	OM984505		OM286920	OM287006	ON013668	ON101523	OM984584	*Archidendronclypearia* (Jack) I.C.Nielsen	*JA05*	Pa Dalih area, Sarawak	115°50'E, 3°40'N
AAU L.AveryanovVH3188	OM984506		OM286921	OM287007	ON013669	ON101524	OM984585	*Archidendronclypearia* (Jack) I.C.Nielsen	*JA15*	Bi Dup mountain system, Vietnam	108°39'E, 12°8'N
CANB 525617.1	OM984507	OM390196; OM390197	OM286922	OM287008	ON013670	ON101525	OM984586	*Archidendronclypearia* (Jack) I.C.Nielsen	*JA95*	East branch of the Avi Avi River. Gulf Province, PNG	146°30'E, 7°44'S
AAU AmbriW838			OM286923	OM287009	ON013671	ON101526	OM984587	*Archidendroncockburnii* I.C.Nielsen	*JA17*	Wanariset research area, Kalimantan Timur	117°‘E, 1°‘S
NY03986843		OM390198	OM286924	OM287010	ON013672	ON101527	OM984588	*Archidendroncontortum* (Mart.) I.C.Nielsen	*T97*	Near Kuala Lumpur, Malaysia	
BRI AQ0380332			OM286925	OM287011				*Archidendronforbesii* Baker f.	*JA38*	Papua New Guinea, Central; Subitana, Sogeri sub-dist., Central, Papua	147°31'E, 9°25'S
BISH752370	OM984509	OM390199	OM286926	OM287012	ON013673	ON101528	OM984589	*Archidendronglabrum* (K.Schum.) Lauterb. & K.Schum.	*JA04*	Siboma, Sayama, track along the ridgeline S from Camp 1. PNG	147.298°E, 7.52857°S
BISH763497	OM984510	OM390200	OM286927	OM287013	ON013674	ON101529	OM984590	*Archidendronglabrum* (K.Schum.) Lauterb. & K.Schum.	*JA115*	Morobe Province; Oomsis, behind PNG Forestry station.	146.821°E, 6.71325°S
BRI AQ0380375			OM286928	OM287014	ON013675	ON101530	OM984591	*Archidendronglandulosum* Mohlenbr. ex Verdc.	*JA39*	Brown River F.R. Central Province, PNG	147°10'33.78"E, 9°30'24.60"S
AAU J.F.Maxwell82-141	OM984511		OM286929	OM287015	ON013676	ON101531	OM984592	*Archidendronglobosum* (Blume) I.C.Nielsen	*JA20*	Near Bukit Kallang, Singapore	
AAU Bjornland445	OM984512		OM286930	OM287016		ON101532	OM984593	*Archidendronglomeriflorum* (Kurz) I.C.Nielsen	*JA10*	Chiang Mai: Amphoe Muang, Mae Rim, Thailand	
CANB 544379.1	OM984516		OM286933	OM287018	ON013679	ON101535	OM984596	*Archidendrongrandiflorum* (Sol. ex Benth.) I.C.Nielsen	*JA100*	Gabba Island, Torres Strait. Queensland, Australia	142°38'22"E, 9°46'8"S
BRI AQ0814833	OM984513; OM984514		OM286931	OM287017	ON013677	ON101533	OM984594	*Archidendrongrandiflorum* (Sol. ex Benth.) I.C.Nielsen	*JA42*	Curramore Sanctuary Nature Reserve, 14km NW of Maleny. Queensland, Australia	152°4'05"E, 26°41'43"S
CNS 131336.1	OM984515		OM286932		ON013678	ON101534	OM984595	*Archidendrongrandiflorum* (Sol. ex Benth.) I.C.Nielsen	*JA43*	Mt Lewis, Carbine Tableland. Queensland, Australia	145°16'E, 16°31'S
MEL 2391892A	OM984517; OM984518	OM390201	OM286934	OM287019	ON013680	ON101536	OM984597	*Archidendrongrandiflorum* (Sol. ex Benth.) I.C.Nielsen	*Z109*	Atherton Arboretum. Tag #846. Queensland, Australia	145°29'8.6"E, 17°15'31.4"S
AAU Kostermans22121			OM286935	OM287020	ON013681	ON101537		*Archidendronharmsii* Malm	*JA74*	Mbengen, West Flores	
AAU H.M.Christensen279	OM984519	OM390202; OM390203	OM286936	OM287021	ON013682	ON101538	OM984598	*Archidendronhavilandii* (Ridl.) I.C.Nielsen	*JA75*	Pa Dalih area, Sarawak	115°50'E, 3°40'N
CANB 596487.1	OM984521	OM390206	OM286939	OM287024	ON013685	ON101541	OM984600	*Archidendronhendersonii* (F.Muell.) I.C.Nielsen	*JA103*	Greenfield Road, Lennox Head. New South Wales, Australia	153°36'E, 28°49'’S
MEL 2293327A	OM984520	OM390204; OM390205	OM286937	OM287022	ON013683	ON101539	OM984599	*Archidendronhendersonii* (F.Muell.) I.C.Nielsen	*JA44*	Brandy Creek Road, 9 km S of Airlie Beach. Queensland, Australia	148°43'15"E, 20°21'2"S
QRS 18805.2			OM286938	OM287023	ON013684	ON101540		*Archidendronhendersonii* (F.Muell.) I.C.Nielsen	*JA45*	Between Starcke homestead and Starcke River. Queensland, Australia	145°5'E, 14°55'S
MEL 2391969A	OM984522	OM390207; OM390208	OM286940	OM287025	ON013686	ON101542	OM984601	*Archidendronhendersonii* (F.Muell.) I.C.Nielsen	*Z114*	Cairns, cultivated in garden. Queensland, Australia	145°46'15"E, 16°55'13"S
QRS 117169.1	OM984523	OM390209	OM286941	OM287026	ON013687	ON101543	OM984602	*Archidendronhirsutum* I.C.Nielsen	*JA46*	Claudie River. Queensland, Australia	143°15'E, 12°44'S
CNS 142441.1	OM984524	OM390210	OM286942	OM287027	ON013688	ON101544	OM984603	*Archidendronhirsutum* I.C.Nielsen	*JA86*	Umagico, Cape York. Queensland, Australia	142°21'E, 10°53'19"S
MEL 2391887A	OM984525	OM390211	OM286943	OM287028	ON013689	ON101545	OM984604	*Archidendronhirsutum* I.C.Nielsen	*Z113*	Atherton Arboretum. Tag #482. Queensland, Australia	145°29'8.6"E, 17°15'31.4"S
BISH760310	OM984526	OM390212	OM286944	OM287029	ON013690	ON101546	OM984605	*Archidendronhispidum* (Mohlenbr.) Verdc.	*JA02*	Northern Province; Sibium Mountains; W of Akupe Camp, along Afase River. PNG	148.269°E, 9.28974°S
AAU R.Geesink7254	OM984527		OM286945	OM287030	ON013691	ON101547	OM984606	*Archidendronjiringa* (Jack) I.C.Nielsen	*JA12*	Kao Chong Botanical Garden, Thailand	99°45'E, 7°40'N
BRI AQ0738090	OM984528; OM984529	OM390213	OM286946	OM287031	ON013692	ON101548	OM984607	*Archidendronkanisii* R.S.Cowan	*JA47*	Oliver Creek. Queensland, Australia	145°26'E, 16°8'S
MELUD113392a	OM984530		OM286947	OM287032	ON013693	ON101549	OM984608	*Archidendronkanisii* R.S.Cowan	*JA65*	Shore of creek, end of Stonewood Road, Queensland, Australia	145.40497°E, 16.16685°S
MELUD113385a	OM984531	OM390214	OM286948	OM287033	ON013694	ON101550	OM984609	*Archidendronkanisii* R.S.Cowan	*JA66*	Shore of creek, end of Stonewood Road, Queensland, Australia	145.40497°E, 16.16685°S
BRI AQ0733240	OM984532							*Archidendronkanisii* R.S.Cowan	*Z49*	NPR133, Daintree, Oliver Creek, Queensland, Australia	145°26'29.997"E, 16°8'11.708"S
AAU I.Cowley110			OM286949	OM287034	ON013695	ON101551	OM984610	*Archidendronkinabaluense* (Kosterm.) I.C.Nielsen	*JA76*	Melilas. Ulu Belait, Brunei	
AAU H.M.Christensen1719			OM286950	OM287035	ON013696	ON101552	OM984611	*Archidendronkunstleri* (Prain) I.C.Nielsen	*JA07*	near Nanga Sumpa, Sarawak	112°10'E, 1°20'N
KUN 0599659	OM984533; OM984534	OM390215	OM286951	OM287036	ON013697	ON101553	OM984612	*Archidendronlaoticum* (Gagnep.) I.C.Nielsen	*JA77*		
BRI AQ0835639	OM984535		OM286952	OM287037	ON013698	ON101554		*Archidendronlovelliae* (F.M.Bailey) I.C.Nielsen	*JA48*	Great Sandy National Park; Cooloola Section, Freshwater Road. Queensland, Australia.	153°6'52"E, 25°57'01S
BRI AQ0636343		OM390216; OM390217	OM286953	OM287038	ON013699	ON101555	OM984613	*Archidendronlovelliae* (F.M.Bailey) I.C.Nielsen	*Z112*	Harry’s Hut Road, Cooloola National Park.Queensland, Australia	153°03'E, 25°26'S
MEL 2034578A	OM984536	OM390218; OM390219	OM286954	OM287039	ON013700	ON101556	OM984614	*Archidendronlucyi* F.Muell.	*JA49*	Indooroopilly, cultivated. Queensland, Australia	
MELUD113387a	OM984537		OM286955	OM287040	ON013701	ON101557	OM984615	*Archidendronlucyi* F.Muell.	*JA62*	Lake Road near Cairns, Queensland, Australia	145.6693°E, 16.875165°S
MELUD113393a	OM984538		OM286956	OM287041	ON013702	ON101558	OM984616	*Archidendronlucyi* F.Muell.	*JA63*	Lake Road near Cairns, Queensland, Australia	145.6693°E, 16.875165°S
MELUD113391a	OM984539		OM286957	OM287042	ON013703	ON101559	OM984617	*Archidendronlucyi* F.Muell.	*JA68*	Cape Tribulation Road, adjacent to Coconut Beach resort, Queensland, Australia	145.45726°E, 16.11345°S
MEL 2391968A	OM984540	OM390220	OM286958	OM287043	ON013704	ON101560	OM984618	*Archidendronlucyi* F.Muell.	*Z108*	Cairns, cultivated in garden. Queensland, Australia	145°46'15"E, 16°55'13"S
BISH760584	OM984541		OM286959	OM287044	ON013705	ON101561	OM984619	*Archidendronmegaphyllum* Merr. & L.M.Perry	*JA03*	Central Province; Mt Gerebu, trail towards summit ridge. PNG	147.646°E, 9.46595°S
AAU H.M.Christensen1282			OM286960	OM287045		ON101562	OM984620	*Archidendronmicrocarpum* (Benth.) I.C.Nielsen	*JA06*	Near Sumpa, Sarawak.	112°10'E, 1°20'N
BRI AQ0499073	OM984544	OM390221; OM390222	OM286962	OM287047	ON013707	ON101564	OM984622	*Archidendronmuellerianum* (Maiden & R.T.Baker) I.C.Nielsen	*JA112*	Big Scrub Flora Reserve, NNE of Lismore, New South Wales, Australia	153°19'44.880"E, 28°38'18.228"S
BRI AQ0763292	OM984542; OM984543		OM286961	OM287046	ON013706	ON101563	OM984621	*Archidendronmuellerianum* (Maiden & R.T.Baker) I.C.Nielsen	*JA50*	Tallebudgera Creek Road, reveg site. Queensland, Australia	153°21'57"E, 28°10'37"S
BISH752405	OM984545; OM984546	OM390223	OM286963	OM287048	ON013708	ON101565	OM984623	*Archidendronparviflorum* Pulle	*JA01*	Morobe Province; Siboma, Sayama, above Sayama Creek, to E Camp 1. PNG	147.302°E, 7.52557°S
MEL 2074350A			OM286964	OM287049	ON013709			*Archidendronpellitum* (Gagnep.) I.C.Nielsen	*JA34*	N. de Dalat, prov. Ht. Donnai. Indochina: Annam. Vietnam	108°27'E, 11°57'N
Bell Museum 913425 (WP-3A0575)	OM984547; OM984548	OM390224	OM286965	OM287050	ON013710	ON101566	OM984624	*Archidendronptenopum* Verdc.	*JA116*	Wanang village, Madang, PNG	145°10.631'E, 5°14.238'S
AAU C.Charoenphol5025	OM984549; OM984550		OM286966	OM287051	ON013711	ON101567	OM984625	*Archidendronquocense* (Pierre) I.C.Nielsen	*JA13*	Ko Rang Yai, Thailand	102°23'E, 11°48'N
MEL 2391884A	OM984557	OM390228	OM286969	OM287053	ON013717	ON101573	OM984630	*Archidendronramiflorum* (F.Muell) Kosterm.	*Z111*	Atherton Arboretum. Tag #1652. Queensland, Australia	145°29'8.6"E, 17°15'31.4"S
MELUD113388a	OM984551		OM286967	OM287052	ON013712	ON101568	OM984626	*Archidendronramiflorum* (F.Muell) Kosterm.	*JA67*	Regeneration plot, Daintree Rainforest Observatory, Queensland, Australia	145.45004°E, 16.10268°S
BRI AQ0485087		OM390225	OM286968					*Archidendronramiflorum* (F.Muell) Kosterm.	*Z110*	Shiptons Flat. Queensland, Australia	145°14'E, 15°47'S
AAU Balgooy6769	OM984552	OM390226	OM286970	OM287054	ON013713	ON101569		*Archidendron* sp. nov. in obs.	*JA85*	Pulan Baun, Aru Island Indonesia	134°35'E, 6°30'S
BRI AQ0052837	OM984553; OM984554		OM286971	OM287055	ON013714	ON101570	OM984627	*Archidendronsyringifolium* (Kosterm.) I.C.Nielsen	*JA41*	Agu River branch of the middle Fly River, PNG	141.166667°E, 6.966667°S
MEL 2041191A	OM984555	OM390227	OM286972	OM287056	ON013715	ON101571	OM984628	*Archidendronvaillantii* (F.Muell) F.Muell.	*JA51*	Cape Tribulation, Queensland, Australia	145°27'E, 16°6'15"S
BRI AQ0558405	OM984556		OM286973	OM287057	ON013716	ON101572	OM984629	*Archidendronvaillantii* (F.Muell) F.Muell.	*JA52*	Along Paluma Dam Road, Ethel Creek, Queensland, Australia	146°10'40.222"E, 19°0'7.863"S
MEL 2196304A	OM984558		OM286974	OM287058	ON013718	ON101574	OM984631	*Archidendronwhitei* I.C.Nielsen	*JA53*	State Forest 310 Gadgarra. Queensland, Australia	145°43'26"E, 17°18'13"S
BRI AQ0824396		OM390229	OM286975	OM287059	ON013719	ON101575	OM984632	*Archidendronwhitei* I.C.Nielsen	*JA54*	7km W of Babinda, Queensland, Australia.	145°54'30"E, 17°20'30"S
KUN 0599686	OM984559; OM984560		OM286976	OM287060	ON013720	ON101576	OM984633	*Archidendronxichouense* (C.Chen & H.Sun) X.Y.Zhu	*JA84*	China	
BRI AQ0611431			OM286978	OM287062	ON013723			*Archidendropsisbasaltica* (F.Muell.) I.C.Nielsen	*Z218*	On Isaac River and Hill Creek. 25km S of Glenden, Queensland, Australia	148°7'E, 21°33'01"S
MEL 0290000A			OM286977	OM287061				*Archidendropsisbasaltica* (F.Muell.) I.C.Nielsen	*Z44*	Bladensburg National Park, S of Winton, Poison Paddock. Queensland, Australia	143°2'23"E, 22°41'9"S
MEL 2333247A	OM984561; OM984562		OM286979	OM287063	ON013721	ON101577	OM984634	*Archidendropsisgranulosa* (Labill.) I.C.Nielsen	*Z362*	Prov. Sud, near Yate, north side of Yate River. New Caledonia	166°56'0"E, 22°9'29"S
BRI AQ0430532			OM286980	OM287064	ON013724			*Archidendropsislentiscifolia* (Benth.) I.C.Nielsen	*Z122*	c. 5km north of Kone, south of Kafeate. New Caledonia.	164.78333°E, 21.05°S
MEL 2095888A			OM286981	OM287065	ON013725	ON101578	OM984635	*Archidendropsisthozetiana* (F.Muell.) I.C.Nielsen	*JA144*	Palmgrove National Park, 5km W of Daydream Hill, Queensland, Australia	149°13'29"E, 24°59'3"S
BRI AQ0771148			OM286982	OM287066	ON013722			*Archidendropsisxanthoxylon* (C.T.White & W.D.Francis) I.C.Nielsen	*Z121*	Daintree, narrow ridge above Cassowary Creek, off Stewart Creek road, site 69. Queensland, Australia	145°17'46"E, 16°17'56"S
L.1958248	OM984563	OM390230	OM286983	OM287067	ON013726	ON101579	OM984636	*Falcatariamoluccana* (Miq.) Barneby & J.W.Grimes	*JA134*	KPC area, Sebongkok Utara, East Kalimantan, Indonesia.	117°31'59"E, 0°48'0"N
CANB 367091.1	OM984564	OM390231	OM286984	OM287068	ON013727	ON101580	OM984637	*Falcatariatoona* (F.M.Bailey) Gill.K.Br., D.J.Murphy & Ladiges	*JA149*	Near Earlando, 27 km N of Proserpine. Queensland, Australia	148°33'E, 20°10'S
MEL 1615244A	OM984567	OM390234; OM390235	OM286987	OM287071	ON013730		OM984640	*Pararchidendronpruinosum* (Benth.) I.C.Nielsen	*Z50*	Palm Tree Creek, W of Mt Whitestone township, Queensland, Australia	152°4'E, 27°39'S
CNS 134531.1	OM984565	OM390232	OM286985	OM287069	ON013728	ON101581	OM984638	*Pararchidendronpruinosum* (Benth.) I.C.Nielsen	*JA55*	CSIRO Arboretum, Queensland, Australia	145°29'6"E, 17°15'28"S
QRS 121813.1	OM984566	OM390233	OM286986	OM287070	ON013729	ON101582	OM984639	*Pararchidendronpruinosum* (Benth.) I.C.Nielsen	*JA56*	Clarke Range, Queensland, Australia	148°31'E, 21°16'S,
MEL 2183015A	OM984568; OM984569	OM390236	OM286988	OM287072	ON013731	ON101583	OM984641	*Paraseriantheslophantha* (Willd.) I.C.Nielsen	*Z43*	Merrimu Reservoir, Victoria, Australia	144°29'23"E, 37°38'3"S
BRI AQ0408829	OM984570; OM984571	OM390237	OM286989	OM287073	ON013732	ON101584	OM984642	*Serianthesnelsonii* Merr.	*JA143*	Atop Sailigai Hulo, Rota. Northern Mariana Islands.	145°12'53"E, 14°09'03"N
MEL 2333248A	OM984572	OM390238	OM286990	OM287074	ON013733	ON101585	OM984643	*Serianthespetitiana* Guillaumin	*Z361*	Prov. Sud, near Prony, New Caledonia	166°49'52"E, 22°19'4"S
MELU SRA051	OM984573	OM390239	OM286991	OM287075	ON013734	ON101586	OM984644	*Wallaceodendroncellebicum* Koord.	*Z48*	Bogor Botanic Gardens collection Accession: B19610136	

Total genomic DNA (gDNA) was extracted following the CTAB method of Doyle and Doyle (1987) with modifications as per Shepherd and McLay (2011). Isolated gDNA was quantified with a NanoDrop 2000 (ThermoScientific) spectrophotometer and cleaned with a 2.4 M sodium acetate wash. Recalcitrant herbarium material that failed using the CTAB method was extracted using the AccuPrep Stool genomic DNA extraction kit (Bioneer) using the manufacturer’s protocol with some modifications suggested by Schuster (pers. comm.). Only 30 mg of leaf material was used instead of the recommended 100–200 mg. A total of 600 µl of stool lysis buffer (SL) was added to the extraction tube instead of 400 µl, the incubation step was increased to one hour in total, centrifugation was done for 10 minutes at step five, and to maintain equal volumes, 600 µl of binding buffer was added. Two consecutive washes were performed using buffer 1 (W1). The final elution was done by adding 160 µl total elution buffer in two steps (first 60 µl, and then 100 µl) instead of a single elution with 200 µl.

### Marker selection, primer design and library preparation

Eight nuclear markers (low copy genes: AIGP, CYB6, Eif3E, SHMT, RBPCO, UDPG; nrDNA: ITS, ETS) and four chloroplast DNA intergenic spacer regions (*trn*K-*mat*K, *trn*V-*ndh*C, *psb*D-*trn*T, *trn*L-*rpl*32) were assessed for variability between nine individuals spanning the series of *Archidendron* using Sanger sequencing.

PCR reagents, primers and cycling conditions are described in Suppl. material 1 (Johnson and Soltis 1994; Sun et al. 1994; Käss and Wink 1997; Baldwin and Markos 1998; Miller and Bayer 2001; Ariati et al. 2006; Choi et al. 2006; Shaw et al. 2007; Li et al. 2008). PCR products were visualised on a 1.5% agarose gel with Easy ladder I (Bioline) and cleaned with ExoSAP-IT (USB) as per the manufacturer’s protocol. The purified amplicons were sequenced on an AB3730xl sequencer (Thermo Scientific) at the Australian Genome Research Facility, Melbourne. Sequences were aligned in Geneious v.8.1.4 (Biomatters Ltd.) and assessed for variability between the samples. The most variable loci were then used in a targeted amplicon sequencing (TAS) approach (McLay et al. 2021), sequencing pooled amplicons on an Illumina MiSeq. For this, additional internal primers were designed for the five loci that had a total amplicon length greater than 500 bp, in order to produce shorter amplicons that could be fully sequenced using a 500-cycle sequencing kit. These primers were designed using Primer 3 v.2.3.4 (Rozen and Skaletsky 2000) implemented in Geneious v.8.1.4 (Biomatters Ltd.), selecting priming sites in conserved regions across the nine sequenced individuals.

Library preparation followed the two-step PCR process outlined in McLay et al. (2021). The first step used the region-specific primers to amplify each locus individually for each sample. Initial PCR reactions included 1 × MyTaq Buffer (Bioline), 1.2 µl of MgCl_2_ 2.5 M (Bioline, 100 mg mL), 1.2 µl of dimethyl sulfoxide (DMSO, 99.5%; Sigma-Aldrich), 3 µl of each “tailed” primer (10 µM), 0.375 U of MyTaq (Bioline), 100 ng of gDNA, and ultra-pure water to make up for 16 µl volume. Variations in these reactions are noted in Suppl. material 1 for specific loci. Conditions for PCR were based on those of Choi et al. (2006), Shaw et al. (2007), and Ariati et al. (2006) with modifications as required to obtain successful amplifications (Suppl. material 1). To estimate amplicon concentration to decide the volume of PCR product for amplicon pooling, 2.2 µl of PCR product and 2.5 µl of molecular ladder (Easyladder I, Bioline) were run on 1.5% agarose. A total of 120 ng of each nuclear DNA (ncDNA) region PCR product and 20 ng of each chloroplast region PCR product were pooled in the same well of a 96-well plate. The ncDNA were pooled in a higher concentration to account for the possible presence of different alleles. Pooled samples were cleaned with 1.5 × Serapure beads (Rohland and Reich 2012).

The second step used qPCR to add unique Illumina indexing barcodes to each sample for the pooled amplicons. Indexing PCR reactions consisted of 5 µM of each of index primer (McLay et al. 2021), 3 µl of pooled amplicons, 1 × Kapa HiFi ReadyMix (Biosystems) and ultra-pure water to make up a total of 25 µl reaction. Conditions for PCR were 95 °C for 1 min, followed by 13 cycles of 98 °C for 50 sec, 67 °C for 50 sec, and 72 °C for 20 sec, and a final extension at 72 °C for 30 sec. Each sample was then cleaned with 1.4 × Serapure beads and concentrations were quantified using fluorescence in a EnSpire multimode plate reader. In total, 10 ng of each indexed and cleaned sample was pooled together. The final pooled library was cleaned with 1.5 × Serapure bead-to-sample ratio and the library was submitted to the Australian Genome Research Facility, Melbourne for sequencing on an Illumina MiSeq using a 500 cycle MiSeq v2 Nano Kit.

### ﻿Data analysis

Sequences obtained by Sanger sequencing were aligned by individual locus in Geneious v.8.1.4 (Biomatters Ltd.) and a consensus sequence was generated and used as the reference for the reads obtained by TAS. The demultiplexed TAS Illumina MiSeq files were imported into Geneious v.8.1.4. Reads were trimmed to remove adapters and low-quality sequence. The map-to-reference option was selected to map reads for each sample to the different reference loci using High Sensitivity/Medium settings and a minimum mapping quality of 20. A consensus sequence for each locus was generated for each individual with Generate Consensus Sequence (Threshold = 65%, with Ns called if coverage was less than 10). The forward and reverse reads of the low-copy nuclear genes (LCNG) overlapped so it was possible to phase these loci into separate alleles, but this was not possible for the nuclear ribosomal DNA loci (ETS and ITS) as the reads were not overlapping due to unexpected length variation in both of these loci. Alignments of individual consensus sequences for each locus were generated using MUSCLE (Edgar 2004) in Geneious v.8.1.4 and adjusted manually. For each LCNG, samples with multiple alleles were assessed for topological concordance between the different copies using neighbour-joining trees (using the Geneious tree-builder, HKY model) and NeighbourNet networks (SplitsTree4, default settings, Huson and Bryant 2006), to ensure that a conflicting signal was not introduced from distantly related allelic variants (see Suppl. material 2: SHMT network and tree and Suppl. material 3: RBPCO network and tree). Allelic variants within samples were largely concordant with one-another permitting consensus sequences for those samples to be used for subsequent phylogenetic analyses.

Alignments of all nuclear loci (ncDNA; with consensus sequences for LCNG alignments) were analysed individually to explore gene tree topologies in IQ-TREE v.1.6.12 on the web server (http://iqtree.cibiv.univie.ac.at/, Trifinopoulos et al. 2016) with support estimated with 1,000 ultra-fast bootstrap replicates (UFBS) (Minh et al. 2013). After comparing topologies, four ncDNA loci (ETS, ITS, RBPCO, SHMT) were concatenated into a single matrix as no major incongruencies were observed. The combined ncDNA dataset was partitioned into six partitions corresponding to each locus with the ITS region further divided in ITS1, 5.8S and ITS2 for subsequent analyses. IQ-TREE was used to perform maximum likelihood (ML) analyses on the concatenated ncDNA alignment. The analysis was run with the alignment partitioned and allowing ModelFinder (Kalyaanamoorthy et al. 2017) to identify the optimal substitution models for each partition (Table 2). Node support was estimated using 1,000 UFBS. Bayesian Inference (BI) was performed, with the alignment partitioned by locus. The best model of substitution for each partition was estimated with IQ-TREE model selection using the options: selection criteria of Bayesian (BIC), candidate models JC, F81, K80, HKY, SYM, GTR, heterogeneity types I, G, I+G, and the genomic source of nuclear (Table 2). MrBayes v.3.2.7a (Ronquist et al. 2012) was run using the CIPRES Science Gateway (Miller et al. 2010). Two parallel runs each with eight Monte Carlo Markov Chains were run for five million generations, sampling a tree every 1,000 generations and a burn-in of 25%.

A consensus network of the combined ncDNA dataset was constructed in SplitsTree4 (Huson and Bryant 2006) using the last 101 sampled BI trees (edge weights = mean, threshold = 0.05). This method allows for the visualisation of conflict in a set of trees and provides an alternative method of interpretation to a single fixed topology of a consensus tree.

**Table 2.** ncDNA data partitions and best fit substitution models. Models estimated by IQ-TREE model selection and applied for BI.

Partition	Model
ETS	HKY+F+G4
ITS1	GTR+F+G4
5.8S	SYM+I+G4
ITS2	HKY+F+G4
SHMT	HKY+F+G4
RBPCO	K2P+I

All chloroplast (cpDNA) loci were concatenated into a single matrix for phylogenetic analyses. IQ-TREE was used to perform ML analyses on the cpDNA matrix, with the alignment partitioned by locus, using ModelFinder to identify the optimal substitution model for each locus, and support was estimated using 1,000 UFBS replicates. The resulting topology was very poorly supported (though similar groups to the ncDNA phylogeny were discovered within the genus *Archidendron*). To further investigate cpDNA relationships within *Archidendron*, the outgroups were removed, and the IQ-TREE analysis was performed on the reduced dataset. The UFBS replicates were then used to create a consensus network in SplitsTree4 (edge-weights = mean, threshold = 0.20).

### ﻿Pollen morphology of Archidendropsissubg.Basaltica

Pollen size and surface texture are key morphological features differentiating the subgenera of Archidendropsis but one of the three species of subg. Basaltica (*A.xanthoxylon* (C.T. White & W.D. Francis) I.C. Nielsen) was not examined by Nielsen et al. (1983b). To fill this gap and ensure consistency of results with published data, pollen from *A.xanthoxylon* (BRI AQ0199126, BRI AQ0874091, BRI AQ0199129 and BRI AQ0648303) and *A.basaltica* (F. Muell.) I.C. Nielsen (BRI AQ1003764, BRI AQ0199029, BRI AQ0625292 and BRI AQ0648454) of subg. Basaltica was examined. Pollen grains were obtained from flowers of herbarium specimens under a Zeiss dissecting microscope at the Queensland Herbarium (BRI) using clean forceps and a fine brush. Samples were mounted on aluminium stubs using double-sided carbon tabs and coated with gold using an Agar Scientific Automatic Sputter Coater. Pollen grains were observed and photographed using a Phenom G2 5keV (kiloelectron-volt) desktop scanning electron microscope (PhenomWorld). Pollen diameter for 10 grains of *A.basaltica* and eight grains of *A.xanthoxylon* was measured using ToupView (TOUPTEK PHOTONICS) software; overall fewer grains were available on specimens of *A.xanthoxylon* for microscopy.

## ﻿Results

### ﻿Targeted amplicon sequencing loci

Of the eight nuclear loci only four were included in the final phylogenetic analyses: SHMT, RBPCO, ITS and ETS. ETS and ITS amplified well, were variable, and are commonly used phylogenetic markers in Caesalpinioideae phylogenetic studies. Of the LCNGs, SHMT was the most informative, followed by RBPCO; allelic variation was found in some individuals for all LCNGs. Exploring allelic variation in the SHMT (36 samples with alleles) and RBPCO (24 samples with alleles) showed that for samples with more than one allele, the copies were closely related to each other (Suppl. material 2: SHMT network and tree and Suppl. material 3: RBPCO network and tree). Two LCNGs were excluded because few individuals of the target genera were successfully sequenced; only 12 sequences of *Archidendron* and two sequences of *Archidendropsis* were obtained for AlGP, and only 16 sequences of *Archidendron* and one *Archidendropsis* were obtained for Eif3E. The remaining two LCNG loci (CYB6 and UDPG) are not included in the analyses due to their short lengths, 240 bp and 202 bp respectively, and lack of variation.

Of the four chloroplast loci, *trn*K-*mat*K was the most informative, followed by *psb*D-*trn*T and then *trn*V-*ndh*C. However, only one of the three blocks of *trn*V-*ndh*C was successfully sequenced. The internal primers designed allowed 100% coverage for the *trn*K-*mat*K, 81% coverage for the *psb*D-*trn*T, and less than 30% coverage for the *trn*V-*ndh*C. It was not possible to obtain sequences for all samples for all blocks in which the three cpDNA regions were divided; as a result the cpDNA dataset was patchy. The *trn*L-*rpl*32 intergenic spacer did not amplify well, with 10 samples partially sequenced, and it was not included in final analyses.

### ﻿Phylogenetic analyses

**Figure 2.**
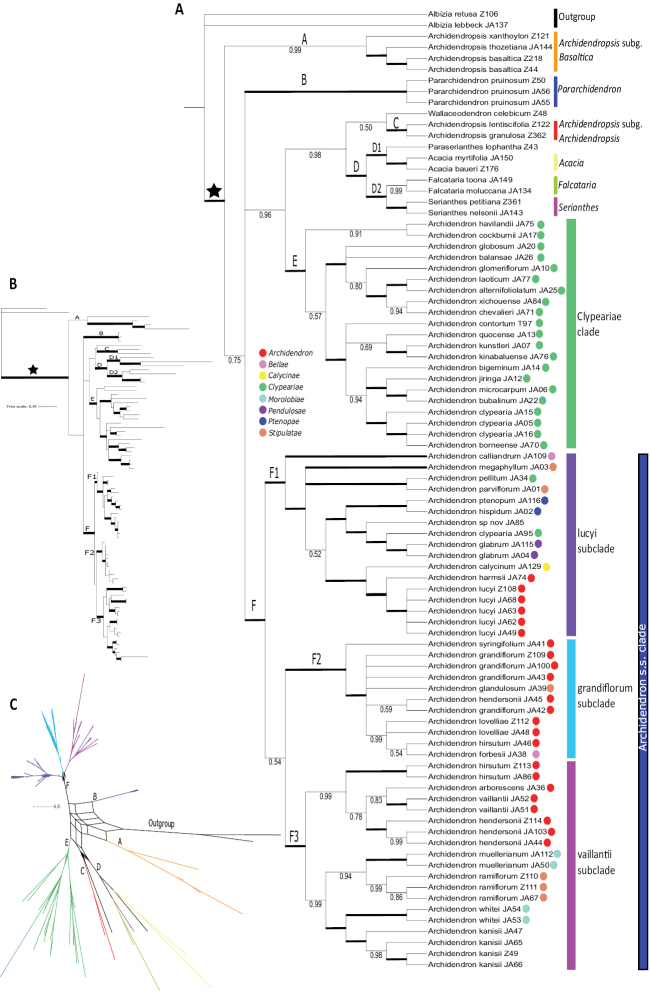
Combined ncDNA phylogeny of the Archidendron clade. The Bayesian Inference (BI) cladogram, phylogram, and consensus network for the combined ncDNA dataset are presented **A** Cladogram: the star indicates the Archidendron clade sensu Koenen et al. (2020). Nodes with PP = 1.0 are shown in bold while other nodes with PP ≥ 0.50 are noted under the node. Clades are labelled with letters above the node. Coloured bars to the right of clades are names discussed in the text. Nielsen’s series of *Archidendron* are shown as coloured circles next to the sample name; key to colour and series in legend **B** Phylogram: clades are labelled as per **A** and nodes with a PP = 1.0 are shown in bold **C** Consensus network: branches are colour coded and labelled as per the clades of **A**.

The topologies of the combined ncDNA Bayesian and IQ-TREE analyses were congruent (nodes supported with UFBS ≥ 95; PP ≥ 0.90) and the Bayesian tree is presented (Fig. 2A,B). The Archidendron clade was recovered as monophyletic (PP 1.0) with six well supported clades (A–F) resolved within it. However, the relationships between clades A–F were not well resolved or supported with a polytomy in the backbone of the phylogeny. Clade A (PP 0.99) includes all three species of Archidendropsissubg.Basaltica, clade B (PP 1.0) includes the three samples of *Pararchidendronpruinosum* (Benth.) I.C. Nielsen, and clade C (PP 1.0) includes the two sampled representatives of Archidendropsissubg.Archidendropsis. Four monophyletic genera are grouped together in clade D (PP 1.0), with *Acacia* sister to *Paraserianthes* in clade D1 (PP 1.0) and *Falcataria* sister to *Serianthes* (PP 1.0) in clade D2 (Fig. 2A). Clade E (PP 1.0) comprises all but two sampled representatives of Archidendronser.Clypeariae, and all other samples of *Archidendron* are placed in clade F (PP 1.0). Clades C, D and *Wallaceodendron* are related (PP 0.98) and together are sister to Clade E (PP 0.96; Fig. 2A).

Within *Archidendron*, only one of Nielsen’s eight series is resolved as monophyletic (ser. Ptenopae) within subclade F1 (Fig. 2A). Clade E, the Clypeariae clade had two main lineages and several smaller supported subclades within them. Clade F, the Archidendron s.s. clade is segregated into three well supported subclades: the lucyi subclade (F1, PP 1.0) that includes three fully supported lineages; the grandiflorum subclade (F2, PP 1.0) that is poorly resolved; and the vaillantii subclade (F3, PP 1.0) that comprises two well supported lineages (PP 0.99; Fig. 2A–C).

Of the 12 species of *Archidendron* that included more than one accession, seven are monophyletic (*A.glabrum* (K. Schum.) K. Schum. & Lauterb., *A.kanisii* R.S. Cowan, *A.lucyi* F. Muell., *A.muellerianum*, *A.ramiflorum* (F. Muell.) Kosterm., *A.vaillantii* (F. Muell.) F. Muell. and *A.whitei*), one is unresolved (*A.lovelliae* (F.M. Bailey) I.C. Nielsen), and four are not monophyletic (*A.clypearia* (Jack) I.C. Nielsen, *A.grandiflorum* (Sol. ex. Benth.) I.C. Nielsen, *A.hendersonii* (F. Muell.) I.C. Nielsen and *A.hirsutum* I.C. Nielsen). Three of the four samples of *A.clypearia* form a clade (within clade E, Fig. 2; PP 1.0) with *A.borneense* (Benth.) I.C. Nielsen nested among them. One sample of *A.hendersonii* (JA45) is related to *A.grandiflorum* within clade F2; all other samples of *A.hendersonii* (Z114, JA103, JA44) form a clade within F3 (PP 1.0; Fig. 2A). Another species falling in both subclades F2 and F3 is *A.hirsutum*, with one sample (JA46) related to *A.forbesii* Baker f. and *A.lovelliae* in subclade F2 (PP 0.99), and the other two (Z113 and JA86) forming a sister pair in subclade F3 (PP 1.0; Fig. 2A).

The consensus network of the final 101-sampled BI trees shows the degree of topological uncertainty between the genera in the Archidendron clade (Fig. 2C). While each respective genus is well-supported as monophyletic (except *Archidendropsis* and *Archidendron* as described above) the relationships between the genera are highly uncertain, reflecting the lack of support in the consensus phylogenies. However, the network reinforces the distinction between the two clades of *Archidendropsis*, and the distinction of the Clyperiae clade from the rest of *Archidendron*.

**Figure 3.**
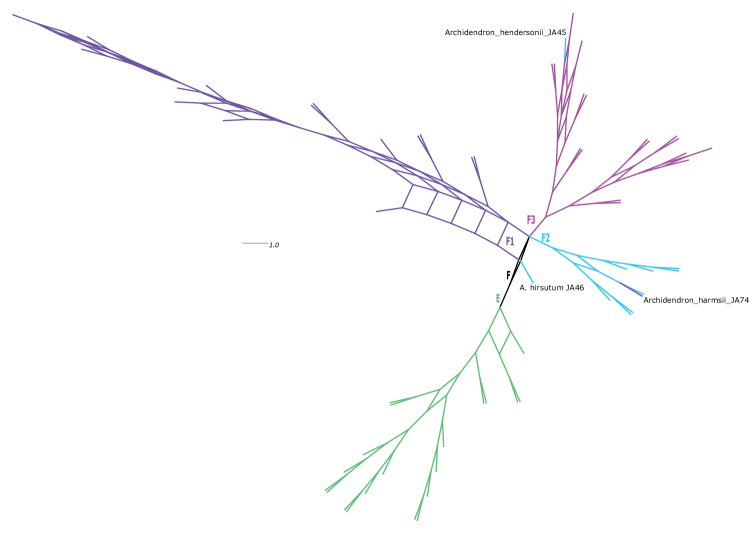
Combined cpDNA consensus network of clades within the genus *Archidendron*. The branches are labelled, and colour coded according to clades in Fig. 2A. Samples that have changed position relative to the ncDNA tree (as discussed in the text) are labelled with their name on the network.

The phylogeny of the three cpDNA loci combined lacks support for nearly all nodes (Suppl. material 4: cpDNA tree). Of the supported nodes there are two that are incongruent with the ncDNA tree (Fig. 2): *Paraserianthes* is sister to *Falcataria* (UFBS 100), and *A.harmsii* Malm is supported in the grandiflorum subclade (UFBS 95) sister to *A.grandiflorum* JA100 (UFBS 97; Suppl. material 4: cpDNA tree). The consensus network of the UFBS replicates (with splits present in at least 20% of trees) reflects the patterns in the ncDNA phylogeny, with four distinct groupings within *Archidendron* (Fig. 3). Within these groupings, several individuals are placed in different clades to the ncDNA tree: *A.hendersonii* JA45 is placed in the vaillantii subclade rather than the grandiflorum subclade, and *A.harmsii* JA74 is in the grandiflorum subclade rather than the lucyi subclade (Fig. 3).

### ﻿Pollen morphology of Archidendropsissubg.Basaltica

**Figure 4.**
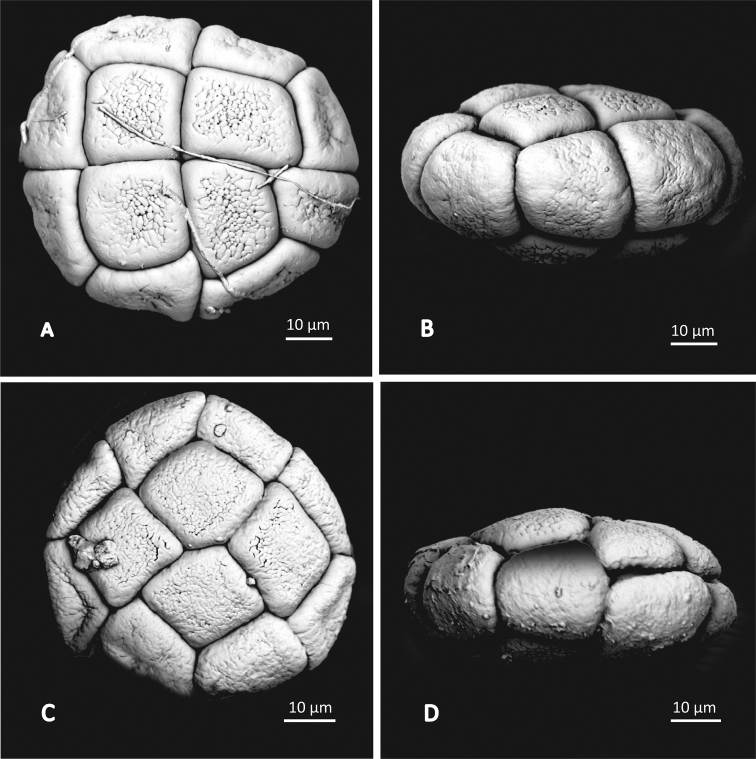
Scanning electron micrographs of Archidendropsissubg.Basaltica pollen. *Archidendropsisxanthoxylon* (**A** BRI AQ0199126 and **B** BRI AQ0874091) and *Archidendropsisbasaltica* (**C** BRI AQ0199029 and **D** BRI AQ01003764).

The pollen measurement results are consistent with Nielsen et al. (1983a, 1983b). The pollen of the two species examined (*A.basaltica* and *A.xanthoxylon*) are aggregated into symmetrical 16-celled polyads with a diameter of 55–62 μm for *A.basaltica* and 62–68 μm for *A.xanthoxylon* (Fig. 4). Fossules were present on the surface of all grains of both species, but they were fainter on the peripheral cells compared to the central ones and overall fainter on *A.basaltica* compared to *A.xanthoxylon* (Fig. 4).

## ﻿Discussion

### ﻿Phylogeny of the Archidendron clade

Our study presents the most taxon-rich sampling of the Archidendron clade of any phylogenetic analyses to date. We confirm that the Archidendron clade sensu Koenen et al. (2020) of Indomalayan-Australasian genera (*Acacia*, *Archidendron*, *Archidendropsis*, *Falcataria*, *Serianthes*, *Pararchidendron*, *Paraserianthes* and *Wallaceodendron*) is robustly supported, yet the relationships between the constituent clades are poorly resolved and lack support. This result is not unexpected given we used only four ncDNA loci and that phylogenomic studies based on hundreds of loci also yield short branches with low support across the backbone of the Archidendron clade (Koenen et al. 2020; Demeulenaere et al. 2022; Ringelberg et al. 2022). It has been suggested that this lack of resolution may be the result of extremely rapid speciation and that the backbone of this clade could be best regarded as a polytomy within the Ingoid legumes (Koenen et al. 2020). The differences in published topologies of the Archidendron clade are illustrated in Demeulenaere et al. (2022) but it is clear that further work based on increased sampling of phylogenomic data is required to uncover the evolutionary history of the clade.

Despite the poorly resolved backbone of the Archidendron clade, many clades within it are robustly supported and corroborate published phylogenies, as well as shedding new light on the genera *Archidendron* and *Archidendropsis* (Fig. 2). Four genera of the Archidendron clade are confirmed to be monophyletic – *Acacia* (Miller and Bayer 2001; Luckow et al. 2003; Miller et al. 2003; Brown et al. 2008), *Falcataria* (Brown et al. 2011), *Pararchidendron* and *Serianthes* (Demeulenaere et al. 2022) – and the previously suggested non-monophyly of *Archidendron* and *Archidendropsis* (Brown et al. 2008, 2011; Iganci et al. 2016; Demeulenaere et al. 2022; Ringelberg et al. 2022) is confirmed and clarified by increased sampling within these genera.

### ﻿Phylogenetic relationships within *Archidendron*

The genus *Archidendron* is not monophyletic, and the eight series, while useful for identification purposes, do not coincide with evolutionary lineages (Fig. 2). The only series confirmed to be monophyletic was series *Ptenopae* from the island of New Guinea, the smallest series comprising just two species with two-winged leaf rachises and pinnae: *A.ptenopum* Verdc. and *A.hispidum* (Mohlenbr.) Verdc. (Nielsen et al. 1984b). The monophyly of series *Calycinae* and *Pendulosae* was not tested, as only one species of each was sampled, however, all other series (*Archidendron*, *Bellae*, *Clypeariae*, *Morolobiae*, and *Stipulatae*) are not monophyletic. *Archidendron* is instead resolved into two well supported lineages, one of which is primarily distributed in western Malesia and mainland Asia (the Clypeariae clade; clade E, Figs 1–3) and the other (the Archidendron s.s. clade; clade F, Figs 1–3) mostly restricted to eastern Malesia and Australia. These two lineages have been identified in previous phylogenetic studies but the sampling for each was extremely limited, with at most seven species of one lineage included (Brown et al. 2008, 2011; Iganci et al. 2016; Demeulenaere et al. 2022; Ringelberg et al. 2022). The further segregation of the Archidendron s.s. clade into three well supported lineages, the lucyi (F1), the grandiflorum (F2), and the vaillantii subclades (F3; Figs 2–3), is novel.

These three subclades of the Archidendron s.s. clade reflect geographic distributions to some extent, but no macromorphological characters have been identified to clearly delineate them. The grandiflorum and vaillantii subclades are predominantly Australian with some southern New Guinean species included, while the lucyi subclade is geographically more broadly distributed in the Lesser Sunda Islands, the Moluccas, through New Guinea to the Solomon Islands with only one species, *A.lucyi*, extending into northern Australia. Morphologically, the lucyi subclade includes all the sampled species lacking stipules that are not from ser. Clypeariae (i.e. *A.calliandrum* de Wit, *A.harmsii*, and *A.glabrum*), although stipules are reported for other species in this clade, three with stipular glands (*A.lucyi*, *A.megaphyllum* Merr. & L.M. Perry, *Archidendron* sp. nov. JA85), two with stipules only (*A.ptenopum* and *A.hispidum*) and *A.parviflorum* Pulle having both stipular glands and stipules (AAU Balgooy 6769; Nielsen et al. 1984b). All sampled species in the grandiflorum and vaillantii subclades have stipules, except *A.arborescens* (Kosterm.) I.C. Nielsen and *A.forbesii*, which have stipular glands (BM000946689; BRI AQ0380081; BRI AQ052589; Nielsen et al. 1984b) The placement of an undescribed species (*Archidendron* sp. nov. JA85) from the Aru Islands (Moluccas) in the lucyi subclade fits the geographic range. Ivan Nielsen noted this as a putative new species in October 1998 (AAU Balgooy 6769) but it does not align with any of the 20 imperfectly known species he outlined (Nielsen et al. 1984b), highlighting that further taxonomic work is required.

Three species in the Archidendron s.s. clade were not resolved as monophyletic (Fig. 2A), although it is unlikely these are issues with species delimitation. The paraphyly of *A.grandiflorum* (Fig. 2), a morphologically consistent species across a large geographic range (Brown pers. obs.), could be the result of potentially rapid and recent divergence or may be due to insufficient phylogenetically informative characters in this study. The latter could also apply to the polyphyletic species (*A.hendersonii* and *A hirsutum*), as *A.hendersonii* JA45, which is placed separately from the other conspecific samples is missing data for two of the four ncDNA loci (Table 1). However, this was not the case for *A.hirsutum* JA46. Re-examination of the vouchers of all accessions of *A.hendersonii* and *A.hirsutum* confirmed their identifications, suggesting that incomplete lineage sorting or paralogy problems associated with one or more nuclear loci could explain these non-monophyletic species; further data are required to investigate this.

The Clypeariae clade (clade E, Figs 2–3) includes all sampled species of ser. Clypeariae (19/51), except one accession of *A.clypearia* (JA95) from Papua New Guinea and *A.pellitum* (Gagnep.) I.C. Nielsen from Vietnam. Series *Clypeariae* was previously recognised in *Pithecellobium* as section Clypearia until Nielsen et al. (1984b) expanded *Archidendron* based on evidence from shared wood anatomy, inflorescence and pod morphology (Nielsen et al. 1984b). Characters of the pods are also useful to differentiate series *Clypeariae* from the rest of *Archidendron*. Nielsen et al. (1984b) described six pod types and most species of ser. Clypeariae have pod type 2 (long funicle, opens ventral suture first) or 6 (straight pods with overgrown seeds), while the other series primarily have pod type 1 (opens dorsal suture first, short funicles). Seeds of ser. Clypeariae are usually flattened and are not embedded in the pericarp, which is possibly linked to characteristics of the pod, such as dryness (de Wit 1942; Nielsen 1981, 1992; Nielsen et al. 1984b). Additionally, the combination of lack of stipules and solitary, stipitate ovaries delineates ser. Clypeariae (Nielsen et al. 1984b). Individually though, these characters are not diagnostic, as some species with sessile ovaries are placed in ser. Clypeariae (e.g. *A.occultatum* (Gagnep.) I.C. Nielsen and *A.turgidum* (Merr.) I.C. Nielsen), other species lacking stipules are placed in series *Archidendron* (e.g. *A.harmsii* and *A.tjendana* (Kosterm.) I.C. Nielsen), and two Philippine species of ser. Clypeariae (*A.apoense* (Elmer) I.C. Nielsen and *A.merrillii* (J.F. Macbr.) I.C. Nielsen) have more than one ovary but both are stipitate (Nielsen et al. 1984b). Given these morphological differences of ser. Clypeariae from the rest of *Archidendron*, together with the non-monophyly of the genus, there are grounds for segregating *Clypeariae* as a distinct genus; however, we are not proposing such a taxonomic change here for several reasons. First, there are many shared morphological characters between species of *Archidendron* s.l.; second, the shallow backbone of the ncDNA tree remains poorly supported with topological uncertainty between lineages; third, the placement of two species of ser. Clypeariae within the Archidendron s.s. clade (clade F; A.clypeariavar.velutinum (Merr. & L.M. Perry) I.C. Nielsen and A.pellitum) raises further doubts; and fourth, phylogenetic sampling of species remains incomplete. All these issues suggest that denser taxon sampling and larger phylogenomic datasets are required before re-classifying *Archidendron* as two genera.

*Archidendronclypearia* is the most widespread species of *Archidendron*, found from India through to Papua New Guinea. The morphological variation within *A.clypearia* has been used to recognise four infraspecific taxa (Legume Phylogeny Working Group 2021): subsp. *clypearia*, subsp.subcoriaceum (Thwaites) M.G. Gangop & Chakrab., var.sessiliflorum (Merr.) I.C. Nielsen, and var.velutinum. The one accession of *A.clypearia* placed outside the Clypeariae clade (JA95) (Fig. 2A) has been identified as var.velutinum (Brown, pers. obs. of CANB525617; previously only identified to species level by the collector), the only infraspecific taxon found in eastern Malesia (Sulawesi, Moluccas and PNG). The three other samples of *A.clypearia* included in the phylogeny have not been assigned to infraspecific taxa but they are not likely var.velutinum, as they are from Malaysia and Vietnam and lack the woolly to velutinous hairs on the lower surface of the leaflets (Brown per. obs.). Taxonomic revision and denser phylogenetic sampling of *A.clypearia* from across its morphological and geographic range is required to verify this placement, delineate the taxa and investigate if var.velutinum should be raised to species level (Merrill and Perry 1942) or if there are intermediate forms as suggested by Kostermans (1966). The only other species of series *Clypeariae* that extends into eastern Malesia, *A.palauense* (Kaneh.) I.C. Nielsen, from the Moluccas through to the Solomon Islands (Nielsen et al. 1984b), was not sampled here. There are no obvious morphological characters that support placement of *A.pellitum* outside the Clypeariae clade, as it has the full combination of diagnostic characters of ser. Clypeariae: compressed pods with a long (3–5 mm) funicle, stipitate single ovary and no visible stipules (US 2515891; P01818442; Nielsen 1981). In addition, no evidence of paralogy in the nuclear loci of *A.pellitum* and A.clypeariavar.velutinum (JA95) was noted in this study; all sequences suggest they fall in the A.lucyi subclade.

The last revision of the genus *Archidendron* (Nielsen et al. 1984b) significantly advanced our understanding of the genus but more detailed taxonomic study is still required, focusing especially on the large number of species known from incomplete material and widespread morphologically variable species, such as *A.clypearia*. To resolve the backbone of the Archidendron clade and inform decisions about generic delimitation to deal with the non-monophyly of *Archidendron*, we recommend further sampling of ser. Clypearia, particularly from the Wallacean region of Malesia (i.e. Moluccas, Sulawesi, Philippines), together with further genomic sampling.

### ﻿Phylogenetic relationships within *Archidendropsis*

While *Archidendropsis* is not monophyletic, its two subgenera (*Archidendropsis* and *Basaltica*) are (Fig. 2). The species within each subgenus have long been recognised as closely related (Bentham 1875; Nielsen 1981) but the two subgenera themselves have not always been associated with each other. For example, Bentham (1875) placed the species of each subgenus in different sections of Albiziabased on inflorescence shape. Species ofsubgenusArchidendropsis that have flowers arranged in cylindrical spikes were placed by Bentham (1875) in AlbiziasectionLophantha Benth. (an illegitimate name later corrected to AlbiziasectionPachysperma (Benth.) Fosberg by Fosberg (1965)). Within this section they were separated from the other taxa, which are now recognised as *Paraserianthes*, into series *Platyspermae* Benth. because they have flattened, broadly orbiculate seeds (Bentham 1875). The two species of subgenus Basaltica known at that time (*A.basaltica* and *A.thozetiana* (F. Muell.) I.C. Nielsen) were placed by Bentham in his large section Eualbizzia distinguished by flowers in globular heads and flattened orbicular seeds (Bentham 1875). Within that section, these taxa were placed into series *Obtusifolia*, which corresponds to the Australian species with 1–2 jugate leaves, ovate, oblong or obtuse leaflets, short petioles, pedunculate heads in the axils, and small sessile flowers.

It was only recently that the species of the two subgenera were united within *Archidendropsis* by Nielsen (1983) based on characters of the fruit and seed: pods dehiscent along both sutures, and seeds that are winged, thin-walled and lack a pleurogram. However, Nielsen himself questioned whether the subgenera should be congeneric, noting that if they were not, “*the evolution of the winged thin walled seeds without pleurogram should have happened twice*” (Nielsen et al. 1983a: p. 337). The results presented here (Fig. 2) alongside two recent phylogenomic analyses (Demeulenaere et al. 2022; Ringelberg et al. 2022) show that the two subgenera of *Archidendropsis* do not form a monophyletic group, suggesting these seed characteristics are indeed the result of convergent evolution.

The presence of a pleurogram is common in mimosoid genera (Gunn 1984), and is considered to have evolved multiple times (Maumont 1993). Within the Archidendron clade, *Archidendron* and *Archidendropsis* are the only two genera whose seeds lack a pleurogram (Nielsen 1992). The absence of a pleurogram has been associated with short-lived ‘recalcitrant’ seeds (i.e. seeds which lack dormancy and can be viviparous; Nielsen 1992) and has been thought to be an adaptive response to humid environments (Corner 1951 in Nielsen 1992; Maumont 1993). Like the absence of a pleurogram, winged seeds are also rare in mimosoids occurring in only eight genera, including *Archidendropsis* (Gunn 1984). The possession of a winged seed has been suggested to be an adaptation for wind-dispersal but there have been no published observations of this in *Archidendropsis* (Gunn 1984; Nielsen 1992). The short viability of *Archidendropsis* seeds has been linked to the restricted geographic ranges of individual species (Nielsen 1983). However, humidity may be a more important determinant of these distributions, as the ranges of the two Australian species occurring in drier, non-rainforest habitats are more than 10 times larger than the rainforest species (e.g. *A.basaltica* ≥ 750,000 km^2^ compared to *A.xanthoxylon* c. 8,750 km^2^ (AVH 2021)). The habitats of *A.basaltica* and *A.thozetiana* are also more open than for *A.xanthoxylon*, but these two species generally have narrower wings on their seeds than the rainforest species *A.xanthoxylon* (Cowan 1998), suggesting that the wing is unlikely to have an impact on wind dispersal. Morphological features that have been used to unite the two subgenera in *Archidendropsis* are thus homoplasious and not useful for generic delimitation.

The non-monophyly and clear morphological distinctions between them means that the two subgenera can no longer be treated as congeneric and need to be placed in separate genera. As the type of *Archidendropsis* (*A.fulgens* (Labill.) I.C. Nielsen) is from subg. Archidendropsis, it is subg. Basaltica that requires a new name. No name exists at the generic level for these taxa, as they have previously been placed in *Acacia*, *Albizia* and *Archidendropsis* (Mueller 1859; Bentham 1875; Fosberg 1965; Nielsen 1983), names which are all typified by other taxa.

**Figure 5.**
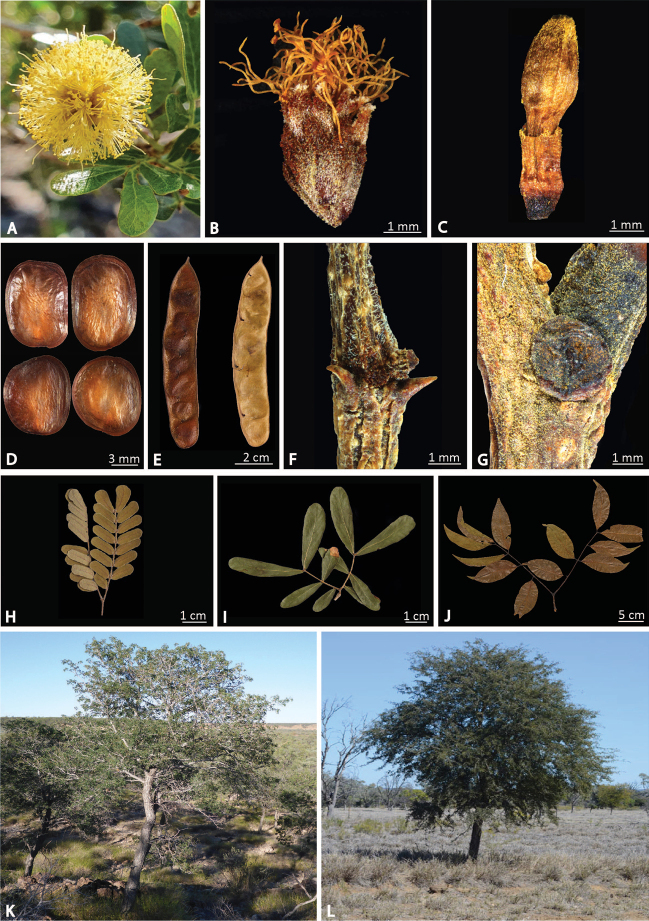
Morphology of *Heliodendron*. Plate showing diagnostic features of the new genus *Heliodendron***A** inflorescence of *H.thozetianum*, Hazelwood Gorge, west of Mackay, Queensland (photo, Stuart Worboys, Australian Tropical Herbarium) **B** single flower of *H.basalticum* (BRI AQ0648454) showing hairs on calyx and corolla **C** mature bud of *H.xanthoxylon* (BRI AQ0874091) showing hairs on the lobes of the calyx and corolla **D** seeds of *H.basalticum* (BRI AQ0746724) **E** overall pod shape of *H.xanthoxylon* (BRI AQ0234095) **F** small rigid stipules of *H.basalticum* (BRI AQ0673898) **G** glandular stipule of *H.xanthoxylon* (BRI AQ0771148). Whole leaf showing overall leaflet size and shape of **H***H.basalticum* (BRI AQ0648454) **I***H.thozetianum* (BRI AQ0611464), and **J***H.xanthoxylon* (BRI AQ0874091). Habit of *H.basalticum* from **K** Bladensberg National Park, Queensland (photo, Dale Richter, Queensland Herbarium) **L** 65 km west south-west of Blackall, Queensland (photo, Murray Fagg, Australian Plant Image Index, Australian National Botanic Gardens).

In addition to the aforementioned morphological differences between the two subgenera, species of subg. Basaltica are endemic to Australia, whereas those of subg. Archidendropsis are found in New Caledonia, New Britain, the Solomon Islands and on the island of New Guinea (Fig. 1B). Furthermore, there are several pollen characters separating the two subgenera (Nielsen et al. 1983a). Pollen of subg. Basaltica has isometric channels in the tectum and is aggregated into smaller polyads (55–68 μm), cf (80–120 μm) for subg. Archidendropsis where the tectum has non-isometric channels (Fig. 4; Nielsen et al. 1983a). The pollen surface of subg. Basaltica has fossules on the central cells, with either faint fossules or smooth peripheral cells, while in subg. Archidendropsis the surface of all pollen cells has small rounded areoles or deep fossules (Fig. 4; Nielsen et al. 1983a). Species of subg. Basaltica have sessile flowers arranged in globular pedunculate heads, rather than in spikes or racemes. Although one species of subg. Archidendropsis, *A.fournieri* (Vieill.) I.C. Nielsen, also has flowers arranged in globular pedunculate heads, it does not share the other diagnostic characters of subg. Basaltica, it is endemic to New Caledonia, its seeds are not winged, and the diameter of the pollen polyads is larger, fitting within the size range for subg. Archidendropsis (Nielsen 1983). Another character noted by Nielsen et al. (1983a) to differentiate the two subgenera, was the shape of the stipules, with those of subg. Basaltica being small and often developed into stipular spines (to 1.2 mm long; Brown pers. obs.; Fig. 5F) that are early caducous. However, the stipules of *A.xanthoxylon* were not recorded by Nielsen et al. (1983a) and are not like other Australian species being 1.2–3 mm long, ovate to triangular, dark gland-like and persistent (Brown, pers. obs., BRI AQ022813, BRI AQ0234095, BRI AQ0771148, BRI AQ199127, BRI AQ0199128; Fig. 5G). These stipules do differ, however, from those of the species of subg. Archidendropsis which, if present, are usually small (c. 1 mm), ovate or filiform and often caducous (Nielsen 1983).

Flowers arranged in globular heads, seeds lacking a pleurogram with a narrow peripheral membranous wing and flat, narrowly oblong, brown pods opening along both sutures distinguish this new genus from other Australian mimosoid legumes, and the keys in Flora of Australia (Cowan 1998) and available on KeyBase (Bean 2021; KeyBase 2021) still remain suitable.

### Taxonomic treatment

#### ﻿ Heliodendron

Taxon classificationPlantaeFabalesLeguminosae

Gill.K. Br. & Bayly gen. nov.

2B4ED609-159C-5CE2-8571-9E5607762628

urn:lsid:ipni.org:names:77303797-1

Fig. 5

##### Diagnosis.

A genus of mimosoid legumes similar to *Archidendropsis* but differing in the following combination of features: inflorescences of glomerules, calyx and corolla with hairs (restricted to the lobes in *H.xanthoxylon*); stipules either small (to 1.2 mm) rigid and caducous or glandular (1.2–3 mm long) and persistent; pollen arranged in polyads diameter of 55–68 μm; pollen tectum with isometric channels. In contrast, *Archidendropsis* has inflorescences of spikes, spiciform racemes, racemes or in one species glomerules, but when in glomerules the calyx and corolla are glabrous; stipules (if present) either small (c. 1mm) ovate or filiform and often caducous, or large auriculate, orbicular, or cordate and persistent; pollen polyad diameter of 80–120 μm, pollen tectum with non-isometric channels.

##### Description.

Trees or shrubs, with terete branchlets. Stipules either resembling small thorns to 1.2 mm long that are early caducous, or persistent circular-ovate glands 1–3 mm in diameter. Leaves bipinnate, pinnae 1–2 pairs with 1.5–11 leaflet pairs per pinna; glands at the junction of pinnae circular or triangular to rhombic, +/- circular glands at the junction of leaflet petiolules. Leaflets opposite, subsessile (0.2–0.7 mm) or long (3.5–7 mm) petiolulate; elliptic to elliptic-lanceolate or oblong, 2–38 mm × 1.5–15 mm, glabrous to puberulous. Inflorescence of globular heads 0.5–1.7 mm in diameter, either simple or arranged into a panicle up to 35 cm long. Flowers: homomorphic, yellow to cream, sessile. Calyx 1.5–3 mm long, tubular to subcampanulate; corolla 2.5–7 mm long, tubular to narrowly campanulate. Ovary 0.8–2 mm long, solitary and shortly stipitate; stamens numerous 5–9 mm long, united basally into a tube that equals or slightly exceeds the corolla tube. Pollen 16-celled polyads with a diameter of 55–68 μm, tectum with isometric channels. Pod brown, valves chartaceous, 6–22 cm × 0.5–2.5 mm, oblong, flat and dehiscing along both sutures. Seeds lacking a pleurogram, flat, circular to ovate or obliquely ovate, 5–13 mm, with a narrow 0.2–1 mm peripheral, membranous wing. Fig. 5.

##### Type.

*Heliodendronbasalticum* (F. Muell.) Gill.K. Br. & Bayly ≡ *Acaciabasaltica* F. Muell., *Journal of the Proceedings of the Linnean Society*, *Botany* 3: 146 (1859)

##### Etymology.

From the Greek *helios* (sun) and *dendron* (tree) alluding to the endemic distribution of the genus in the Australian state of Queensland, widely known as the “sunshine state”, the globular, sun-like inflorescences of yellow flowers, and the tree habit (Fig. 5A, K, L) and also in reference to the genera *Archidendropsis* (in which the species were previous placed) and *Archidendron* (which they resemble).

##### Homotypic synonym.

Archidendropsissubg.Basaltica I.C. Nielsen, *Bulletin du Muséum National d’Histoire Naturelle*. *Section B*, *Adansonia*: *Botanique Phytochimie* 5(3): 325 (1983).

##### Notes.

We have chosen to create a new name for this genus rather than making a new combination based on the name Archidendropsissubg.Basaltica. This is because using the name “Basaltica” at generic rank would require a change of epithet for the most widespread species in the genus under Art. 23.4 of the International Code of Nomenclature for algae, fungi, and plants (Turland et al. 2018). To minimise taxonomic change, and to avoid potential confusion, we would rather that the species retains its well-known epithet, which has been in continuous use since 1859.

The genus includes the following three species, all endemic to Queensland, Australia (Fig. 1B).

#### ﻿ Heliodendron basalticum

Taxon classificationPlantaeFabalesLeguminosae

(F. Muell.) Gill.K. Br. & Bayly comb. nov.

2ABA19F9-2B7A-5BF4-A0B8-465C8D36B716

urn:lsid:ipni.org:names:77303798-1

##### Basionym.

*Acaciabasaltica* F. Muell., *Journal of the Proceedings of the Linnean Society*, *Botany* 3: 146 (1859). ≡ *Albiziabasaltica* (F. Muell.) Benth., *Flora Australiensis* 2: 422 (1864); *Archidendropsisbasaltica* (F. Muell.) I.C. Nielsen, *Bulletin du Muséum National d’Histoire Naturelle*. *Section B*, *Adansonia*: *Botanique Phytochimie* 5(3): 326 (1983).

##### Type.

Peak Downs, *F. Mueller 42* (holotype: MEL 594732A image!; isotype K000822321 image!).

#### ﻿ Heliodendron thozetianum

Taxon classificationPlantaeFabalesLeguminosae

(F. Muell.) Gill.K. Br. & Bayly comb. nov.

DBDDAF57-645B-5E72-A76E-BFC43E16744E

urn:lsid:ipni.org:names:77303799-1

##### Basionym.

*Acaciathozetiana* F. Muell., *Fragmenta Phytographiae Australiae* 4(24): 9 (1863). ≡ *Albiziathozetiana* (F. Muell.) F. Muell. ex Benth., *Flora Australiensis* 2: 422 (1864); *Archidendropsisthozetiana* (F. Muell.) I.C. Nielsen, *Bulletin du Muséum National d’Histoire Naturelle*. *Section B*, *Adansonia*: *Botanique Phytochimie* 5(3): 326 (1983).

##### Type.

Fort Cooper, [*A. Thozet?] no. 29*. (Lectotype, designated by R.S. Cowan, *Nuytsia* 11: 13 (1996)): MEL 595338A image!; residual syntypes: MEL 595339A, MEL 595340A, MEL 595342A, MEL 595377A].

#### ﻿ Heliodendron xanthoxylon

Taxon classificationPlantaeFabalesLeguminosae

(C.T. White & W.D. Francis) Gill.K. Br. & Bayly comb. nov.

645F8512-B16D-5E54-AA5A-0A8389874408

urn:lsid:ipni.org:names:77303800-1

##### Basionym.

*Albiziaxanthoxylon* C.T. White & W.D. Francis, *Proceedings of the Royal Society of Queensland* 41: 141, t. X (1929). *Archidendropsisxanthoxylon* (C.T. White & W.D. Francis) I.C. Nielsen, *Bulletin du Muséum National d’Histoire Naturelle*. *Section B, Adansonia*: *Botanique Phytochimie* 5(3): 326 (1983).

##### Type.

Atherton District, North Queensland, *Overseer brothers s.n.* (Provisional Forestry Board), end of October, 1927 (Lectotype, designated by I.C. Nielsen as “Type”, *Bulletin du Muséum National d’Histoire Naturelle*. *Section B*, *Adansonia*: *Botanique Phytochimie* 5(3): 341 (1983): BRI AQ022813! [2 sheets]; isolectotypes: DNA D0053218 image!, K000822329 image!, MEL 1562403A image!).

##### Notes.

The protologue of *Albiziaxanthoxylon* (White and Francis 1929) gave a location, collector name and month of the collection but did not indicate the herbarium in which the type was held, thus meaning that all specimens of this gathering could be considered syntypes. However, it appears that Nielsen inadvertently typified this taxon, according to Art. 7.11 of the ICN (Turland et al. 2018), when providing the description for the new combination of *Archidendropsisxanthoxylon* with the text “*Type*: *Overseer Brothers*, *Australia*, *N. Queensland*, *Atherton District*, *Oct 1927*, *fl. fr.* (*holo*-,*BRI*; *iso-K*)” (Nielsen et al. 1983a: p. 341). We believe this satisfies the requirements of Art. 7.11 to effectively lectotypify the name, which means that the BRI specimen is the lectotype and the K specimen is the isolectotype. Interestingly, the material illustrated in the protologue is clearly the isolectotype at K, as it is the only type specimen of *Heliodendronxanthoxylon* with pods, and the structure of the inflorescence and leaves is almost identical (K000822329; White and Francis 1929).

In Flora of Australia, Cowan (1998) cited BRI as holding an isotype as well as the holotype of this taxon; however, the two sheets have the same collection details, are labelled as sheet 1 of 2 and sheet 2 of 2, and share a single accession number (BRI AQ022813). Therefore, it is herein determined that these are the one collection, and both represent the holotype (now lectotype; BRI AQ022813).

## ﻿Conclusion

We present the most densely sampled phylogeny of the genera *Archidendron* and *Archidendropsis* to date and confirm that both genera are not monophyletic. The well supported clades within the Archidendron clade based on four nuclear markers agree with more data-rich phylogenomic data sets now being generated. A new genus, *Heliodendron*, endemic to Queensland (Australia), is described for the Australian members of the former Archidendropsissubg.Basaltica. Further sampling of species from subg. Archidendropsis would be beneficial, particularly to ascertain the relationships of the globular flowered *A.fournieri* and the non-New Caledonian representatives of *Archidendropsis* s.s. While *Archidendron* is also not monophyletic, no nomenclatural changes are made, because low phylogenetic support and high topological uncertainty between genera of the Archidendron clade mean that the relationships between the two clades of *Archidendron* remain uncertain. In addition, discrete macromorphological characters need to be identified to distinguish the two lineages of *Archidendron* as the basis for generic re-delimitation. A taxonomic revision of the widespread polymorphic *A.clypearia* would aid this, as our results indicate var.velutinum from eastern Malesia may represent a distinct species. Phylogenomic data and additional sampling of this species would be beneficial before taxonomic changes are made.

## Supplementary Material

XML Treatment for Heliodendron

XML Treatment for Heliodendron basalticum

XML Treatment for Heliodendron thozetianum

XML Treatment for Heliodendron xanthoxylon

## Supplementary materials

Supplementary material 1 Primer sequences and PCR variations Data type Pdf file. Explanation note The reference for the primer and their PCR conditions are provided, along with the variations for PCR reagents and cycling conditions for the initial PCR in the two-step PCR process. * only used for sanger sequencing so no variations to note. Standard PCR reagents prior to variation consisted of 2X QIAGEN PCR buffer (QIAGEN), 5 mM of each dNTP (Bioline), 1 µl of each primer (10 µM), 1.25 µl of dimethyl sulfoxide (DMSO, 99.5%; Sigma-Aldrich), 1 U of Taq DNA polymerase, 100 ng of template and made up to 25 µl with ultra pure water per reaction. Reagent variations, A: not varied; B: 200 ng DNA, 1.2 µl BSA instead of DMSO; C: 200 ng DNA; D: 200ng DNA, 6 μM each primer, 1.5 µl MgCl2, 0.9 µl DMSO and 0.1 µl Taq; E: 6 µM each primer. Cycle variations: Z: 94 °C for 15 mins; 30 cycles of 94 °C for 20 sec, 61 °C for 20 sec, 72 °C for 2 mins; 72 °C for 5 mins; Y: 94 °C for 15 mins; 35 cycles of 94 °C for 20 sec, 61 °C for 20 sec, 72 °C for 2 mins; 72 °C for 5 mins; X: 94 °C for 15 mins; 35 cycles of 94 °C for 20 sec, 55 °C for 30 sec, 72 °C for 2 mins; 72 °C for 7 mins; W: 94 °C for 15 mins; 40 cycles of 94 °C for 20 sec, 50 °C for 1 min, 72 °C for 3 mins; 72 °C for 7 mins; V: 80 °C for 5 mins; 40 cycles of 95 °C for 1 min, 50 °C for 1 min with 0.3 °C/sec ramp, 65 °C for 4 mins; 65 °C for 5 mins; U: 94 °C for 5 mins; 30 cycles of 94 °C for 30 sec, 53 °C for 30 sec, 72 °C for 1 min; 72 °C for 7 mins; T: 80 °C for 5 mins; 30 cycles of 95 °C for 1 min, 50 °C for wwith 0.3 °C/sec ramp, 65 °C for 4 mins; 65 °C for 5 mins. https://binary.pensoft.net/file/733436This dataset is made available under the Open Database License (http://opendatacommons.org/licenses/odbl/1.0/). The Open Database License (ODbL) is a license agreement intended to allow users to freely share, modify, and use this Dataset while maintaining this same freedom for others, provided that the original source and author(s) are credited.Gillian K. Brown, Javier Aju, Michael J. Bayly, Daniel J. Murphy, Todd G.B. McLay

Supplementary material 2 SHMT network and tree Data type Pdf file. Explanation note Neighbour-joining tree and NeighbourNet network are presented with individual samples with more than one allele coloured to highlight their positions. The samples are coloured the same in both the tree and network. The clades that are congruent with Fig. 2 (B, C, D, D1, D2, F1, F2) are labelled. The sequences from species of *Albizia* (Z106, JA137) were removed as they occur on a very long branch relative to the rest of the samples in the network. https://binary.pensoft.net/file/733437This dataset is made available under the Open Database License (http://opendatacommons.org/licenses/odbl/1.0/). The Open Database License (ODbL) is a license agreement intended to allow users to freely share, modify, and use this Dataset while maintaining this same freedom for others, provided that the original source and author(s) are credited.Gillian K. Brown, Javier Aju, Michael J. Bayly, Daniel J. Murphy, Todd G.B. McLay

Supplementary material 3 RBPCO network and tree Data type Pdf file. Explanation note Neighbour-joining tree and NeighbourNet network are presented with individual samples with more than one allele coloured to highlight their positions. The samples are coloured are the same in both the tree and network. Clade B, which is congruent with Fig. 2 is labelled. https://binary.pensoft.net/file/733438This dataset is made available under the Open Database License (http://opendatacommons.org/licenses/odbl/1.0/). The Open Database License (ODbL) is a license agreement intended to allow users to freely share, modify, and use this Dataset while maintaining this same freedom for others, provided that the original source and author(s) are credited.Gillian K. Brown, Javier Aju, Michael J. Bayly, Daniel J. Murphy, Todd G.B. McLay

Supplementary material 4 cpDNA tree Data type Pdf file. Explanation note IQ-Tree of combined cpDNA loci, with all UFBS values shown. The two clades that are congruent with of Fig. 2 are labelled (A and F). Arrows indicate the placement of the two supported incongruences mentioned in the results text. https://binary.pensoft.net/file/733439This dataset is made available under the Open Database License (http://opendatacommons.org/licenses/odbl/1.0/). The Open Database License (ODbL) is a license agreement intended to allow users to freely share, modify, and use this Dataset while maintaining this same freedom for others, provided that the original source and author(s) are credited.Gillian K. Brown, Javier Aju, Michael J. Bayly, Daniel J. Murphy, Todd G.B. McLay
