# Supplementary figures and images for: ﻿Phylogeny and classification of the Australasian and Indomalayan mimosoid legumes Archidendron and Archidendropsis (Leguminosae, subfamily Caesalpinioideae, mimosoid clade)
Source: PhytoKeys. 2022 Aug 22;205:299–333. doi: 10.3897/phytokeys.205.79381 (PMC9848999; doi:10.3897/phytokeys.205.79381)

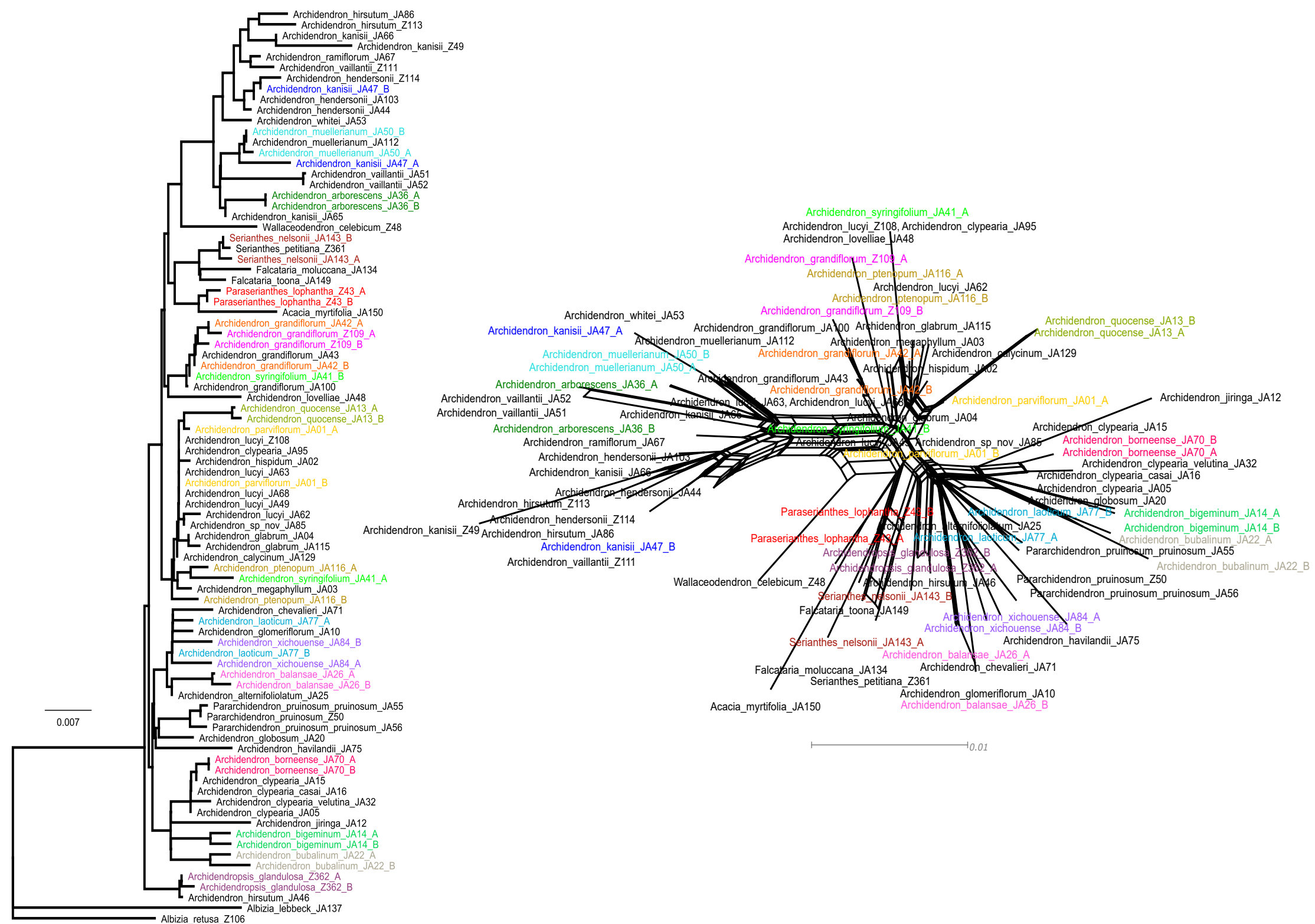

Supplement: Supplementary material 1 — Primer sequences and PCR variations [file phytokeys-205-299_article-79381__-s001.pdf]

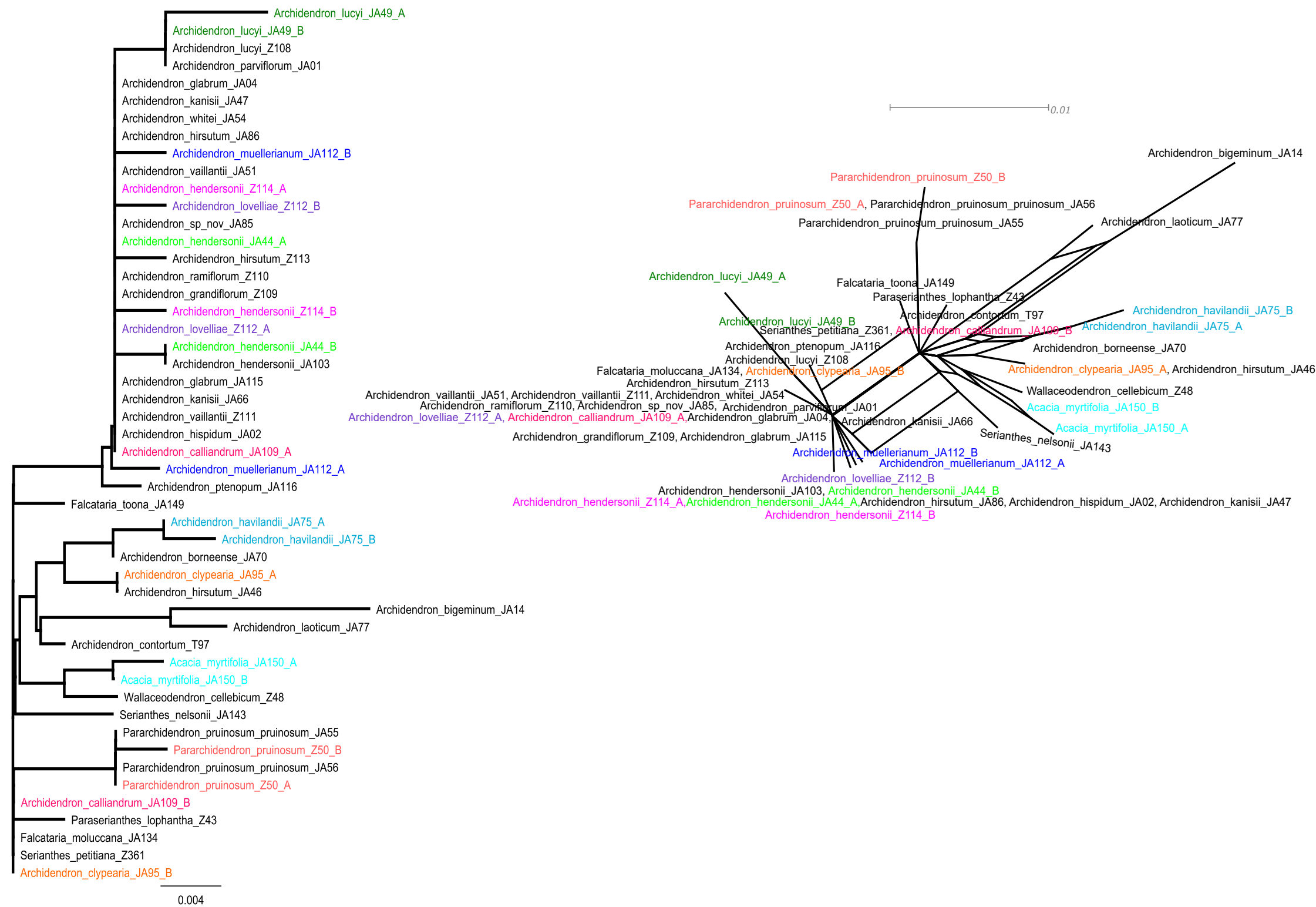

Supplement: Supplementary material 2 — SHMT network and tree [file phytokeys-205-299_article-79381__-s002.pdf]

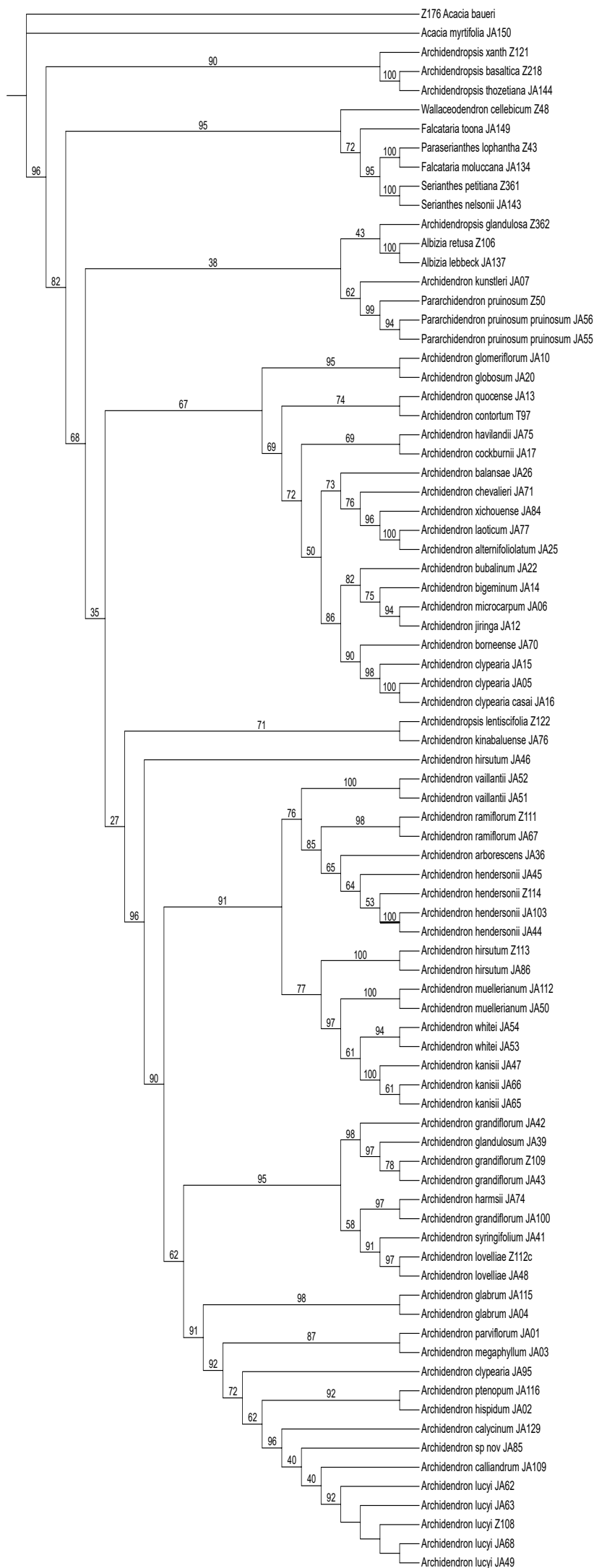

Supplement: Supplementary material 3 — RBPCO network and tree [file phytokeys-205-299_article-79381__-s003.pdf]

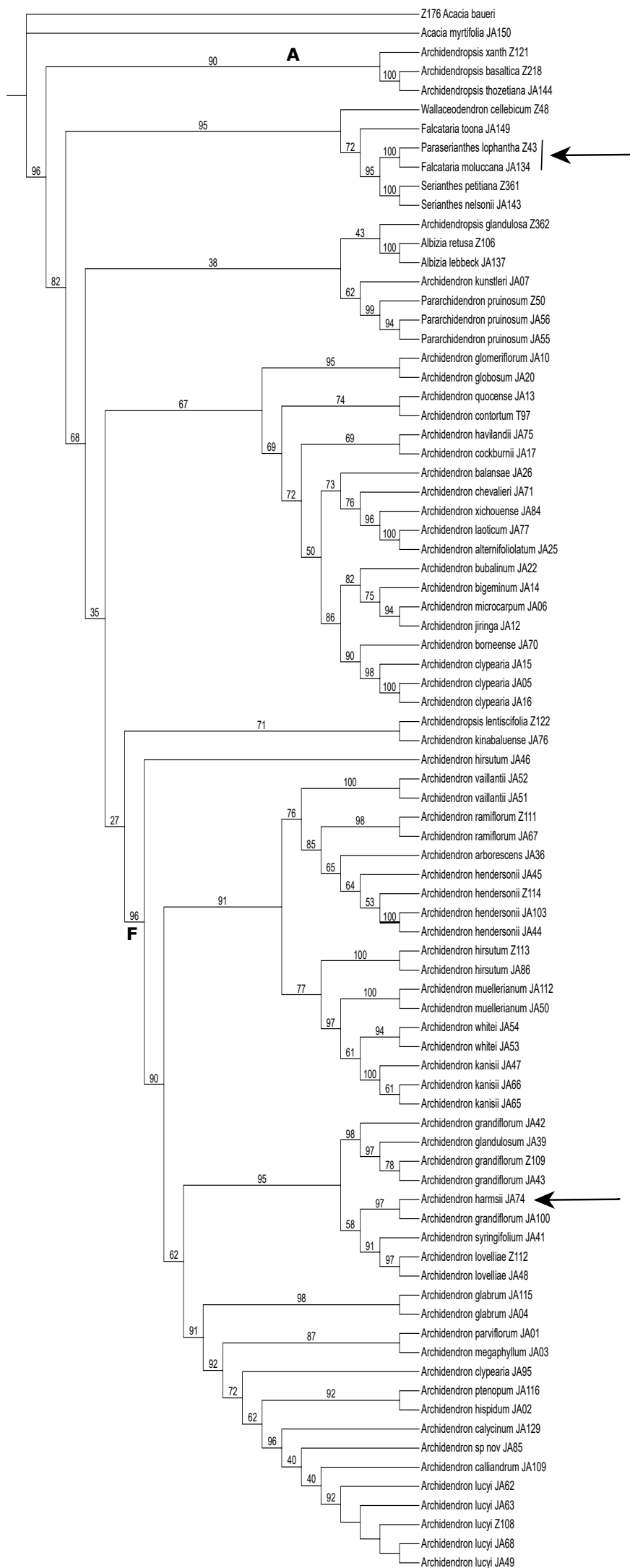

Supplement: Supplementary material 4 — cpDNA tree [file phytokeys-205-299_article-79381__-s004.pdf]
